# Revision of *Anadimonia* Ogloblin, support for synonymy with *Trichocerophysa* Gressitt & Kimoto (Coleoptera, Chrysomelidae, Galerucinae, Galerucitae), with descriptions of one new genus and nine new species

**DOI:** 10.3897/zookeys.1275.179136

**Published:** 2026-03-30

**Authors:** Chi-Feng Lee, Jan Bezděk

**Affiliations:** 1 Applied Zoology Division, Taiwan Agricultural Research Institute, Taichung 413, Taiwan Mendel University in Brno Brno Czech Republic; 2 Fisheries and Hydrobiology, Department of Zoology, Mendel University in Brno, Zemĕdĕlská 1, 613 00 Brno, Czech Republic Applied Zoology Division, Taiwan Agricultural Research Institute Taichung Taiwan

**Keywords:** *
Camphora
*, food plant, Lauraceae, leaf beetles, *
Litsea
*, *
Machilus
*, taxonomy

## Abstract

The synigonymy of *Anadimonia* Ogloblin, 1936 with *Trichocerophysa* Gressitt & Kimoto, 1963 is supported. *Trichocerophysa latifascia* Gressitt & Kimoto, 1963, **syn. nov**. is regarded as a new synonym of *Anadimonia potanini* Ogloblin, 1936. A new genus *Anatrimonia***gen. nov**. is described. *Atysa albofasciata* Jacoby, 1892 and *Trichocerophysa hainana* Gressitt & Kimoto, 1963 (resurrected from synonymy with *T. latifascia* Gressitt & Kimoto, 1963) are transferred to *Anatrimonia***gen. nov**. Nine new species of *Anatrimonia***gen. nov**. are described as follows: *A. cheni***sp. nov**., *A. chungi***sp. nov**., *A. huangi***sp. nov**., *A. jungchani***sp. nov**., *A. meihuai***sp. nov**., *A. tsoui***sp. nov**., *A. wangi***sp. nov**., *A. yuae***sp. nov**., and *A. yunnanensis***sp. nov**.

## Introduction

The genera *Anadimonia* Ogloblin, 1936 and *Trichocerophysa* Gressitt & Kimoto, 1963 are rare and little-known galerucine chrysomelids. *Anadimonia* was established by Ogloblin (1936) for the single species *A. potanini* Ogloblin, 1936 from Sichuan, China. Gressitt and Kimoto (1963) created the genus *Trichocerophysa* for two new species, *T. latifascia* Gressitt & Kimoto, 1963 and *T. hainana* Gressitt & Kimoto, 1963. Kimoto (1989) synonymized *Trichocerophysa* with *Anadimonia* and synonymized *T. hainana* with *T. latifascia*. Medvedev (2005) transferred *Atysa albofasciata* Jacoby, 1892 to *Trichocerophysa*. No subsequent taxonomic works on these genera have been published.

*Trichocerophysa hainana* was firstly recorded from Taiwan by Kimoto (1969). This species was recorded from Taiwan again (Kimoto 1991) but identified as *Anadimonia latifascia* based on a previous synonymy. No other subsequent records have been published. Surprisingly, no specimens were found in the historical collection at the Taiwan Agricultural Research Institute among numerous specimens of other recently revised genera such as *Euphitrea* Baly, 1875 (Lee 2025a) and *Batophila* Foudras, 1860 (Lee 2025c). Fortunately, more than 200 specimens are now available for study from extensive collections made by the Taiwan Chrysomelid Research Team (TCRT) (Lee 2025b). The true species diversity and distributions can now be presented based on adequate material.

## Materials and methods

Adults of this genus can be collected by sweeping leaves of food plants with extendable insect nets, as done for adults of *Taiwanaenidea* Kimoto, 1984 (Lee and Beenen 2015). Adults of some species were also attracted to light traps. In addition, a number of adults were collected during the fogging program conducted by Prof. Man-Miao Yang (楊曼妙) entitled “Fogging a subtropical forest of Central Taiwan: arboreal ground-beetle (Coleoptera, Carabidae) assemblages across an altitudinal gradient”, that was supported by the National Science and Technology Council. The fogging was conducted on two trees, one representing *Machilus zuihoensis* Hayata (15 m in height and 91.2 cm in width), 24°05'50.02"N, 121°02'16.51"E, at Huisunlinchang (惠蓀林場), altitude 1460 m, and the other representing *Neolitsea variabillima* (Hayata) Kaneh. & Sasaki (20 m in heigh and 253 cm in width), 24°04'31.32"N, 121°07'44.80"E, altitude 1450 m (both Lauraceae).

For taxonomic study, abdomens of adults were separated from the forebodies and boiled in 10% KOH solution, followed by washing in distilled water to prepare genitalia for illustrations. The genitalia were then dissected from the abdomens, mounted on slides in glycerin, and studied and drawn using a Leica M165 stereomicroscope. For detailed examinations, a Nikon ECLIPSE 50i microscope was used.

At least three males and females from each species were examined to delimit variability of diagnostic characters. For species collected from more than one locality or with color variations, at least one pair of each sex from each locality and color morph was examined. Length was measured from the anterior margin of the eye to the elytral apex, and width at the greatest width of the elytra. Nomenclature for morphological structures of adults follows Duckett and Daza (2004). Names of plant species follow the Taiwan Encyclopedia of Life (2025). Specimens studied herein are deposited at the following institutes and collections:

**BPBM** Bernice P. Bishop Museum, Hawaii, USA [Keith Arakaki];

**CAS** California Academy of Sciences, California, USA [Christopher C. Grinter, Rachel Diaz-Bastin];

**JBCB** Jan Bezděk collection, Brno, Czech Republic;

**HHCR** Hans Hebauer’s collection, Rain, Germany;

**KMNH** Kitakyushu Museum of Natural History and Human History, Kitakyushu, Japan [Yûsuke Minoshima];

**MCSN** Museo Civico di Storia Naturale “Giacomo Doria”, Genova, Italy [Roberto Poggi];

**NHMB** Naturhistorisches Museum, Basel, Switzerland [Matthias Borer];

**NHMUK** The Natural History Museum, London, UK [Michael F. Geiser, Maxwell V. L. Barclay];

**NMNS** National Museum of Natural Science, Taichung, Taiwan [Bao-Cheng Lai];

**NMPC** National Museum, Prague, Czech Republic [Lukáš Sekerka];

**RBCN** Ron Beenen’s collection, Nieuwegein, Netherlands;

**ZIN** Zoological Institute, Russian Academy of Science, St. Petersburg, Russia [Alexey G. Moseyko].

Exact label data are cited for all type specimens of described species; a double slash (//) divides the data on different labels and a single slash (/) divides the data in different rows. Other comments and remarks are in square brackets: [p] – preceding data are printed, [h] – preceding data are handwritten, [g] – gray label, [r] – red label, [w] – white label.

## Taxonomy

### Anadimonia

Taxon classificationAnimaliaColeopteraChrysomelidae

Ogloblin, 1936

564A6C61-597E-5CAA-91D7-D5C38ECB46EF

Anadimonia Ogloblin, 1936: 127. Type species Anadimonia potanini Ogloblin, 1936, by original designation. Type locality: China.
Trichocerophysa Gressitt & Kimoto, 1963: 471. Type species: Trichocerophysa latifascia Gressitt & Kimoto, 1963, by original designation. Synonymized by Kimoto (1989).

#### Diagnosis.

**Figure 1.**
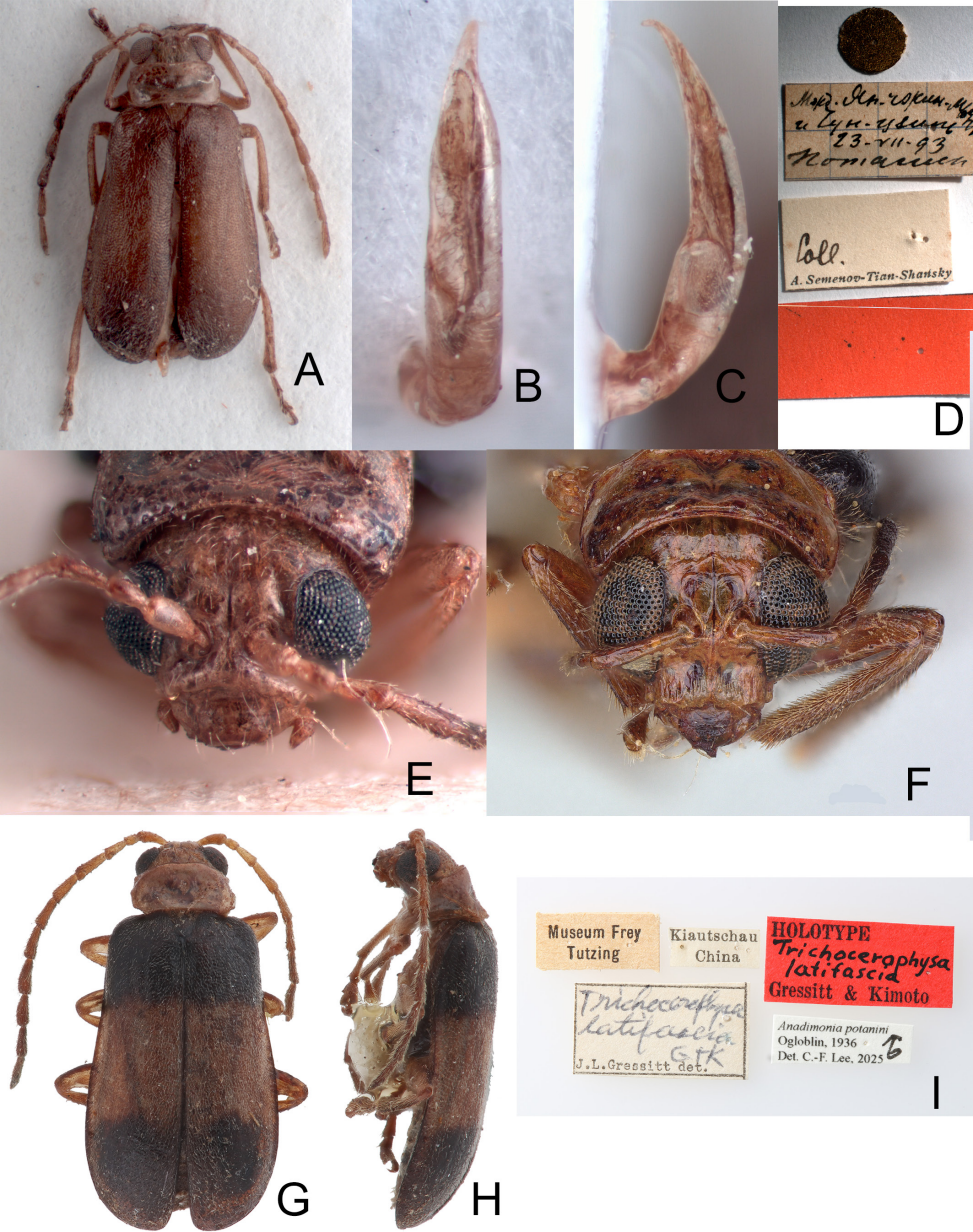
Type specimens and labels **A**. *Anadimonia potanini* Ogloblin, 1936, syntype, dorsal view; **B**. Ditto, aedeagus, dorsal view; **C**. Ditto, aedeagus, lateral view; **D**. Labels pinned with syntype; **E**. Ditto, front view; **F**. *Trichocerophysa hainana* Gressitt & Kimoto, 1963, holotype, front view; **G**. *Trichocerophysa latifascia* Gressitt & Kimoto, 1963, holotype, dorsal view; **H**. Ditto, lateral view; **I**. Labels pinned with holotype.

Adults of *Anadimonia* Ogloblin are similar to those of *Mimastracella* Jacoby & *Anatrimonia* gen. nov. but differ in having the antennal insertions even with anterior margins of the eyes (Fig. 1E) [antennal insertions posterior to anterior margins of eyes in *Anatrimonia* gen. nov. (Fig. 1F)], anterofrontal ridges well developed (Fig. 1E) [anterofrontal ridges reduced in *Anatrimonia* gen. nov. (Fig. 1F)], open anterior coxal cavities (closed anterior coxal cavities in *Anatrimonia* gen. nov.), antennomere III shorter than IV (antennomere III longer than IV in *Mimastracella* and *Anatrimonia* gen. nov.), pronotum with lateral depressions and abbreviated medially (pronotum with lateral depressions interrupted by median groove in *Anatrimonia* gen. nov.), apical margin of last abdominal ventrite with no modification in males (apical margin of abdominal ventrite with median depressions in males of *Mimastracella* and *Anatrimonia* gen. nov.), and asymmetricalalal aedeagus (symmetricalal aedeagus in *Mimastracella* and *Anatrimonia* gen. nov.).

#### Description.

Coloration: generally black, yellow, or white but without metallic color. Body length 4.6–7.0 mm.

Head (Fig. 1E). Labrum trapezoidal, transverse, with six pores in transverse row bearing pale setae, anterior margin medially depressed. Anterior surface of head very short, almost impunctate and glabrous, several setae on anterior margin of clypeus and anterofrontal ridge. Interantennal space narrow, 0.95–1.03 × as wide as diameter of antennal insertion, antennal insertions at level of anterior margins of eyes. Frontal tubercles flat, glabrous; with median longitudinal groove, apically extending into antennal calli, antennal calli visible. Vertex smooth but with dense setae. Antennae filiform, covered with dense setae, antennomere II a little shorter than III, III much shorter than remaining antennomeres; VIII and IX apically widened in males (Fig. 3A), but normal in females (Fig. 3B).

Pronotum 1.95–2.03 × as broad as long, lateral margins slightly rounded, basally narrowed. Disc glabrous, covered with sparse, coarse punctures at sides, with a broad oblique depression at each side, median longitudinal groove not clear. Margined on all sides. Anterior and posterior margins with dense setae, lateral margins without setae. Anterior angles moderately strongly bent downwards, posterior angles rectangular, all angles with setigerous, pore bearing, long, pale setae. Scutellum subtriangular, with dense setae and rounded apex.

Elytra 1.60–1.68 × as long as wide, with extremely dense setae, parallel sided from base to basal 1/4, then rounded, widest at apical 1/4. Humeral calli well developed. Epipleura broad at base, gradually narrowed from basal 1/3, abbreviated near apex. Macropterous.

Ventral surface sparsely covered with fine punctures and pale setae. Anterior coxal cavities widely open. Prosternal process not visible between procoxae. Abdomen simple, posterior margin of last ventrite not modified in both sexes.

Legs slender. Tibiae lacking apical spines. Protarsomeres I not modified in males. Metatarsomere I similar to pro- and mesotarsomeres I, shorter than II and III combined. Tarsal claws bifid in both sexes.

Aedeagus (Fig. 3C) slender, apex asymmetricalalal; internal sac with only one long endophallic sclerite.

Gonocoxae (Fig. 3F) short, tightly conjunct basally; each gonocoxa with eight long setae at apex, apex narrowly rounded, base membranous, with one extremely slender and longitudinal sclerite. Ventrite VIII (Fig. 3E) wide, with dense setae along medial surface of apical margin; disc with dense, tiny setae near medial area. Spermathecal receptaculum (Fig. 3G) strongly swollen; pump wide and curved; sclerotized spermathecal duct wide but short.

#### Included species.

*Anadimonia potanini* Ogloblin, 1936.

### Anadimonia potanini

Taxon classificationAnimaliaColeopteraChrysomelidae

Ogloblin, 1936

EC0408A4-74A4-515A-8A2E-11B9B1DA8139

**Figure 2.**
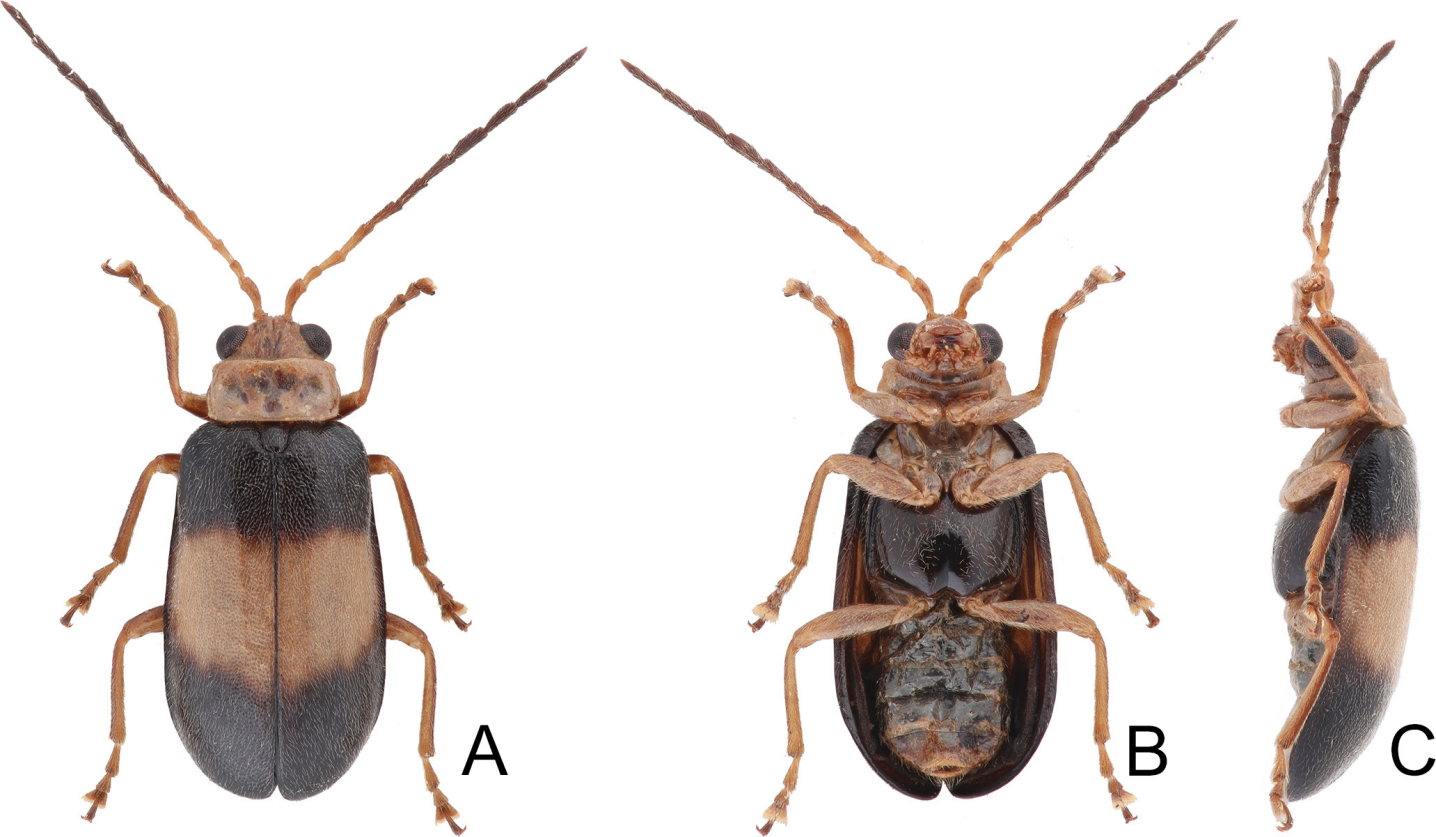
Habitus of *Anadimonia potanini* Ogloblin **A**. Male, dorsal view; **B**. Ditto, ventral view; **C**. Ditto, lateral view.

**Figure 3.**
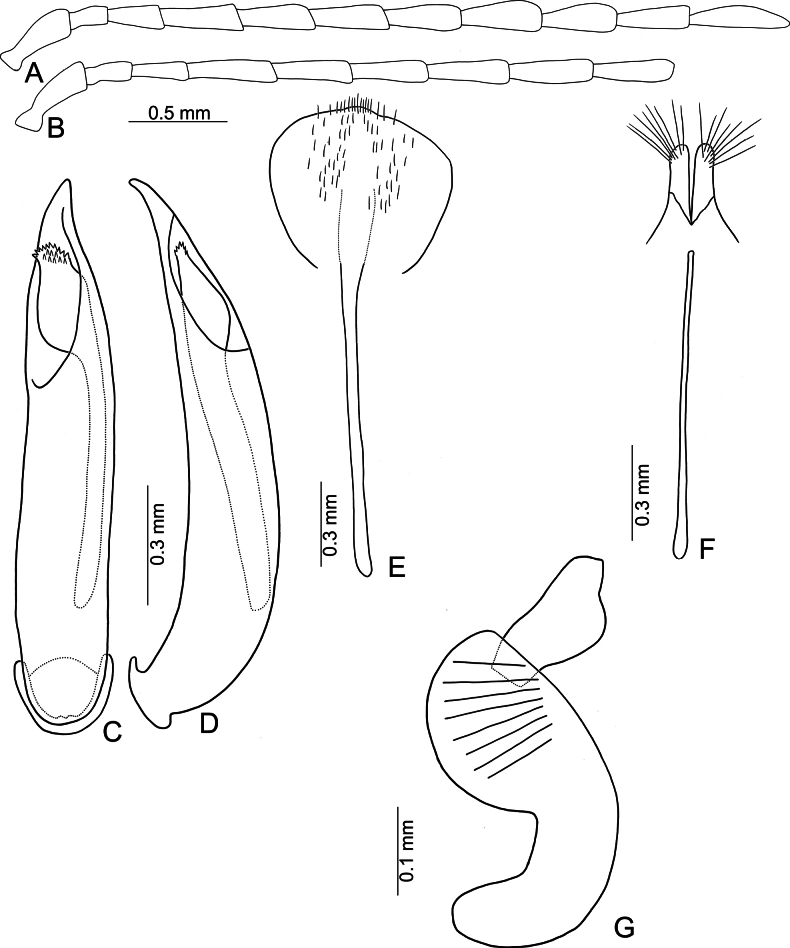
*Anadimonia potanini* Ogloblin **A**. Antenna, male; **B**. Antenna, female; **C**. Aedeagus, dorsal view; **D**. Aedeagus, lateral view; **E**. Abdominal ventrite VIII, female; **F**. Gonocoxae; **G**. Spermatheca.

Figs 1A–E, 1G–I, 2, 3

Anadimonia potanini Ogloblin, 1936: 128 (China: Sichuan).
Trichocerophysa latifascia Gressitt & Kimoto, 1963: 472 (China: Guizhou); Medvedev and Sprecher-Uebersax 1998: 29 (Nepal). Syn. nov.
Anadimonia latifascia : Kimoto 1989: 33 (Vietnam).

#### Type specimen examined.

*Anadimonia potanini*. Several syntypes are deposited in ZIN. One male (Fig. 1A–E) was studied: “(no letters) [gold circle label] // Межд. Ян-чжин-мын [= between Yan-tshin-myn] / и Чун-цзынь [= and Tshun-tsin] / 23-VII-93 / Потанин [= Potanin] // Coll. [h] / A. Semenov-Tian-Shansky [p, w] // (no letters) [red label]”.

*Trichocerophysa latifascia*. ***Holotype*** • (Fig. 1 G–I) (♂, NHMB, by original designation): “Kiautschau / China [p, w] // Museum Frey / Tutzing [p, w] // HOLOTYPE [p] / Trichocerophysa / latifascia [h] / Gressitt & Kimoto [p, r] // Trichocerophysa / latifascia / G & K [h] / J. L. Gressitt det. [p, w]”. ***Paratypes***. • 1♀ (NHMB): “Kiautschau / China [p, w] // Museum Frey / Tutzing [p, w] // ♀ [h, w] // Trichocerophysa / latifascia / Gress. & Kim. [h] / Gressitt & Kimoto det. 196[p]2 [h, w] // ALLOTYPE [p] / Trichocerophysa / S. Kimoto & [h] / J. L. Gressitt [p, r]”; 1♀ (NHMB): “Kiautschau / China [p, w] // Museum Frey / Tutzing [p, w] // PARATYPE [p] / Trichocerophysa / latifascia [h] / Gressitt & Kimoto [p, y] // Trichocerophysa / latifascia / G & K [h] / J. L. Gressitt det. 196[p]1 [h, w]”; 1♂ (BPBM): “Kiautschau / China [p, w] // ♂ [h, w] // spec. ad / Cerophysa [h, w] // Museum Frey / Tutzing [p, w] // PARATYPE [p] / Trichocerophysa / latifasciata [h] / Gressitt & Kimoto [p, y] // Trichocerophysa / latifascia / G & K [h] / J. L. Gressitt det. 196[p]1 [h, w]”; 1♂ (BPBM): “Kiautschau / China [p, w] // ♂ [h, w] // Museum Frey / Tutzing [p, w] // genus / Tricho- [h, w] // N. 111 [h, w] // Trichocerophysa / latifascia / para ♂ G & K [h] / J. L. Gressitt det. [p, w] // PARATYPE [p] ♂ / Trichocerophysa / latifascia [h] / Gressitt & Kimoto [p, y]”; 1♀ (NHMUK): “Kiautschau / China [p, w] // ♂ [h, w] // Museum Frey / Tutzing [p, w] // PARATYPE [p] ♂ / Trichocerophysa / latifascia [h] / Gressitt & Kimoto [p, y] // Para- / type [p, w, circle label with yellow border] // Brit. Mus. / 1963-245. [p, w]”.

#### Additional specimen examined

**(*n* = 13)**. **China** • Fujian: coll. H. Hebauer: 2♂, 4♀ + 5 exs. (HHCR: 2♂ + 5 ex., RBCN: 4♀), Ziyungdongshan, NW slopes, 25°46'N, 117°20'E, 700–1100 m, 29.IV.2008, leg. J. Turna; **Vietnam** • Hoang Lien Son: 1♂ (NHMB), Sa Pa, 11–15.V.1990, leg. V. Kubáň.

#### Diagnosis.

Easily diagnosed based on generic diagnosis since it is monotypic genus.

#### Description.

Length 4.8–6.0 mm, width 2.3–2.7 mm. General color yellowish-brown (Fig. 2); antennae apically darkened of antennomeres VI; scutellum black; elytra black with broad, transverse white band at middle; metathoracic ventrites black, most of abdomen black in males, but two apical abdominal ventrites black in females. Antennae (Fig. 3A) filiform in males, antennomeres VIII–IX apically widened, ratio of lengths of antennomeres I–XI 1.0 : 0.5 : 0.7 : 1.0 : 0.9 : 1.0 : 1.0 : 1.1 : 1.0 : 0.9 : 1.3; ratio of length to width of antennomeres I–XI 2.6 : 2.2 : 2.3 : 2.9 : 2.9 : 3.0 : 2.8 : 2.6 : 2.6 : 3.2 : 4.5; similar in females, but antennomeres VIII and IX normal, ratio of lengths of antennomeres I–IX (X and XI missing) (Fig. 3B) 1.0 : 0.6 : 0.7 : 1.0 : 0.9 : 0.9 : 1.0 : 1.0 : 1.0; ratio of length to width of antennomeres I–IX 2.9 : 2.4 : 3.1 : 4.2 : 3.5 : 3.4 : 3.6 : 3.7 : 3.6. Pronotum 1.95–2.03 × wider than long; lateral margins slightly rounded and basally narrowed, basal margin and apical margin straight; disc with a broad oblique depression at each side, median longitudinal groove obsolete. Elytra 1.60–1.68 × longer than wide; with extremely dense setae, parallel sided from base to basal 1/4, then rounded, widest at apical 1/4. Aedeagus (Fig. 3C, D) slender, ~ 6.3 × longer than wide; lateral margins parallel from base to apical 1/3, apical 1/3 to apex asymmetrical, apex narrowly rounded; slightly and curved near apex and base in lateral view; with one long endophallic sclerite, 0.7 × as long as aedeagus, swollen from apex to apical 1/3, apex with dense teeth. Gonocoxae (Fig. 3F) short, tightly conjunct basally; each gonocoxa with eight long setae at apex, apex narrowly rounded, base membranous, with one extremely slender and longitudinal sclerite. Ventrite VIII (Fig. 3E) wide, with dense setae along medial surface of apical margin; disc with dense tiny setae near medial area; spiculum extremely long. Spermathecal receptaculum (Fig. 3G) strongly swollen; pump wide and curved; sclerotized spermathecal duct wide but short.

#### Variation.

Type specimens of *Anadimonia potanini* have entirely yellowish-brown to brown elytra (Fig. 1A), but specimens in Fujian, China have entirely black elytra.

#### Food plants.

Unknown.

#### Distribution.

China (Sichuan, Guizhou, Fujian), Nepal, and Vietnam.

### Anatrimonia

Taxon classificationAnimaliaColeopteraChrysomelidae

Lee & Bezděk gen. nov.

9827D1AF-93C6-5E45-96DC-D0EB0E605A0C

https://zoobank.org/5756C659-BAEE-4D23-9FF2-E9AD1F89DED5

#### Type species.

*Atysa albofasciata* Jacoby, 1892. Type locality: Myanmar.

#### Diagnosis.

Adults of *Anatrimonia* gen. nov. are similar to those of *Anadimonia* Ogloblin and *Mimastracella* Jacoby, but differ in having the antennal insertions posterior to anterior margins of eyes (Fig. 1F) [antennal insertions even with anterior margins of eyes (Fig. 1E) in *Anadimonia* and *Mimastracella*], anterofrontal ridges reduced (Fig. 1F) [anterofrontal ridges well developed (Fig. 1E) in *Anadimonia* and *Mimastracella*], closed anterior coxal cavities (open anterior coxal cavities in *Anadimonia* and *Mimastracella*), antennomere III longer than IV (antennomere III shorter than IV in *Anadimonia*), pronotum with lateral depressions interrupted by median groove (pronotum with lateral depressions and abbreviated medially in *Anadimonia* and *Mimastracella*), apical margin of abdominal ventrite with median depressions in males (apical margin of last abdominal ventrite with no modification in males of *Anadimonia*), and symmetrical aedeagus (asymmetrical aedeagus in *Anadimonia*).

#### Description.

Coloration: black, white, yellow, and red but without metallic color. Body length 3.5–6.8 mm.

Head. Labrum trapezoidal, transverse, with four pores in transverse row bearing pale setae, anterior margin rounded. Anterior surface of head short, almost impunctate and glabrous, anterofrontal ridge reduced, sparse setae on frons. Interantennal space narrow, 0.75–0.82 × as wide as diameter of antennal insertion, antennal insertions behind anterior margins of eyes. Frontal tubercles flat, glabrous; with median longitudinal groove, apically extending beyond antennal calli, antennal calli indistinct. Vertex smooth but with dense setae. Antennae filiform, covered with dense setae, antennomere II much shorter than III, III longest, shorter than rest of antennomeres; I also extremely long, subequal to III.

Pronotum 1.47–1.95 × as broad as long, lateral margins slightly rounded. Disc glabrous, covered with sparse, coarse punctures at sides, with a broad transverse depression at each side, median longitudinal groove obvious. Margined on all sides. Anterior and posterior margins with dense setae, lateral margins without setae. Anterior angles moderately bent downwards, posterior angles rectangular, all angles with setigerous, pore bearing, long, pale setae. Scutellum subtriangular, with dense setae and rounded apex.

Elytra 1.52–2.02 × as long as wide, with extremely dense setae, parallel sided, subapically convergent. Humeral calli well developed. Epipleura broad at base, gradually narrowed from base, reaching apex. Macropterous.

Ventral surface sparsely covered with fine punctures and setae. Anterior coxal cavities closed. Prosternal process reduced between procoxae. Abdomen simple, posterior margin of last ventrite with median depression in males.

Legs slender. All tibiae lacking apical spines. Protarsomere I not modified in males. Metatarsomere I similar to pro- and mesotarsomeres I, shorter than II and III combined. Tarsal claws bifid in both sexes.

Aedeagus slender, apex symmetrical; internal sac with three endophallic sclerites in most species, shapes of endophallic sclerites variable.

Gonocoxae short, tightly conjunct basally; each gonocoxa with more than ten long setae at apex, apex narrowly rounded, base usually slender. Ventrite VIII wide, with several setae along sides of apical margin; disc without setae. Spermathecal receptaculum strongly swollen; pump wide and curved; sclerotized spermathecal duct wide but short.

#### Etymology.

This new genus name combines *Anadimonia* and *Trichocerophysa* to indicate their close relationship.

#### Included species.

*Anadimonia albofasciata* (Jacoby, 1892), comb. nov., *A. cheni* sp. nov., *A. chungi* sp. nov., *A. hainana* (Gressitt & Kimoto, 1963) stat. res., comb. nov., *A. huangi* sp. nov., *A. jungchani* sp. nov., *A. meihuai* sp. nov., *A. tsoui* sp. nov., *A. wangi* sp. nov., *A. yuae* sp. nov., and *A. yunnanensis* sp. nov.

#### Distribution.

China, India, Myanmar, Laos, Taiwan, and Thailand.

### Anatrimonia albofasciata

Taxon classificationAnimaliaColeopteraChrysomelidae

(Jacoby, 1892) comb. nov.

13620176-6B2C-5F75-BED7-9387814972B9

Figs 4A–C, 5

Atysa albofasciata Jacoby, 1892: 977 (Myanmar).
Trichocerophysa albofasciata : Medvedev 2005: 236.

#### Type specimen examined.

**Figure 4.**
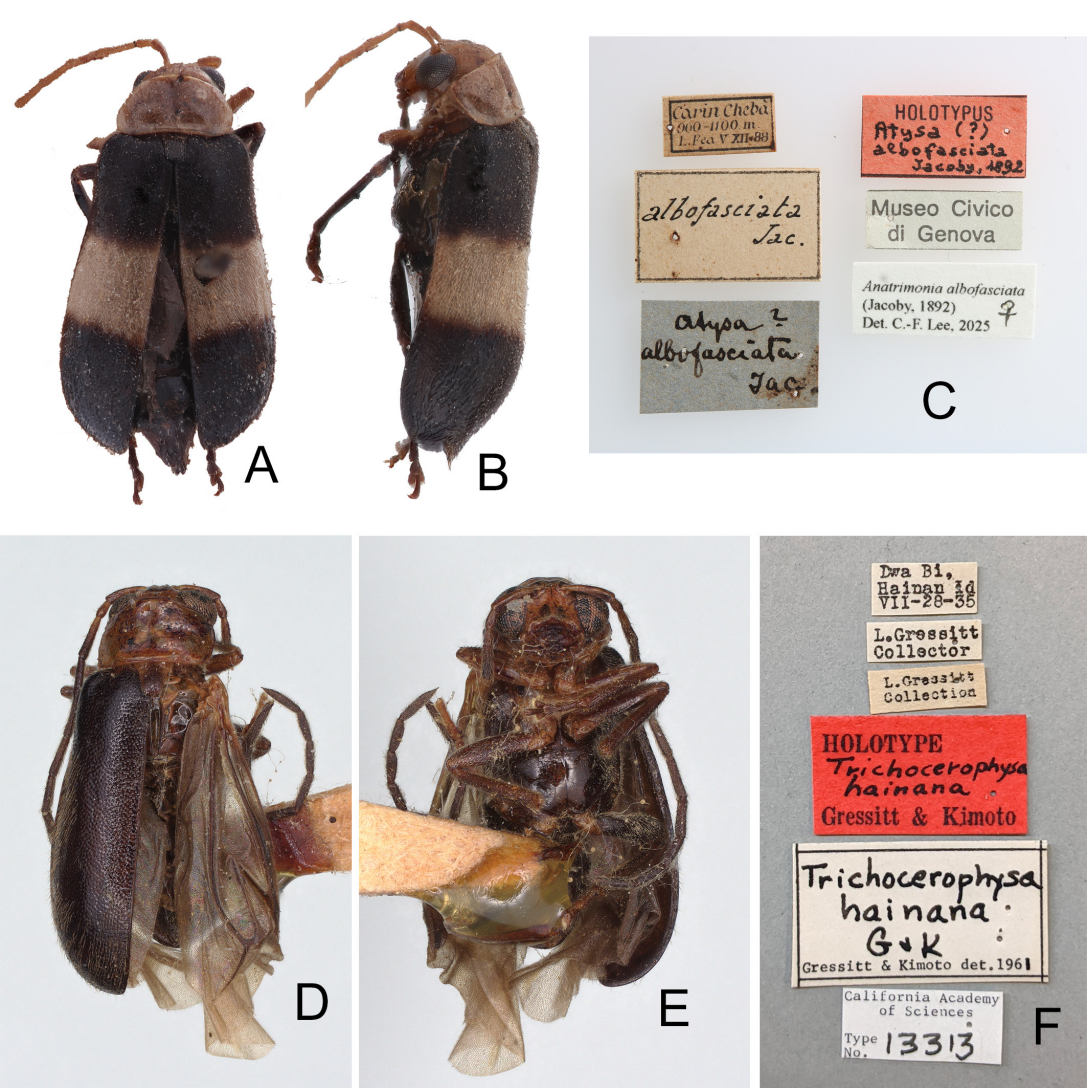
Type specimens and labels **A**. *Atysa albofasciata* Jacoby, 1892, holotype, dorsal view; **B**. Ditto, lateral view; **C**. Labels pinned with holotype; **D**. *Trichocerophysa hainana* Gressitt & Kimoto, 1963, holotype, dorsal view; **E**. Ditto, ventral view; **F**. Labels pinned with holotype.

***Holotype*** • ♀ (Fig. 4A–C) (MCSN, by monotypy): “Carin Chebà / 900–1100 m / L. Fea V VIII-88 [p, w] // albofasciata / Jac. [p, w] // Atysa ? / albofasciata / Jac. [h, g].

**Figure 5.**
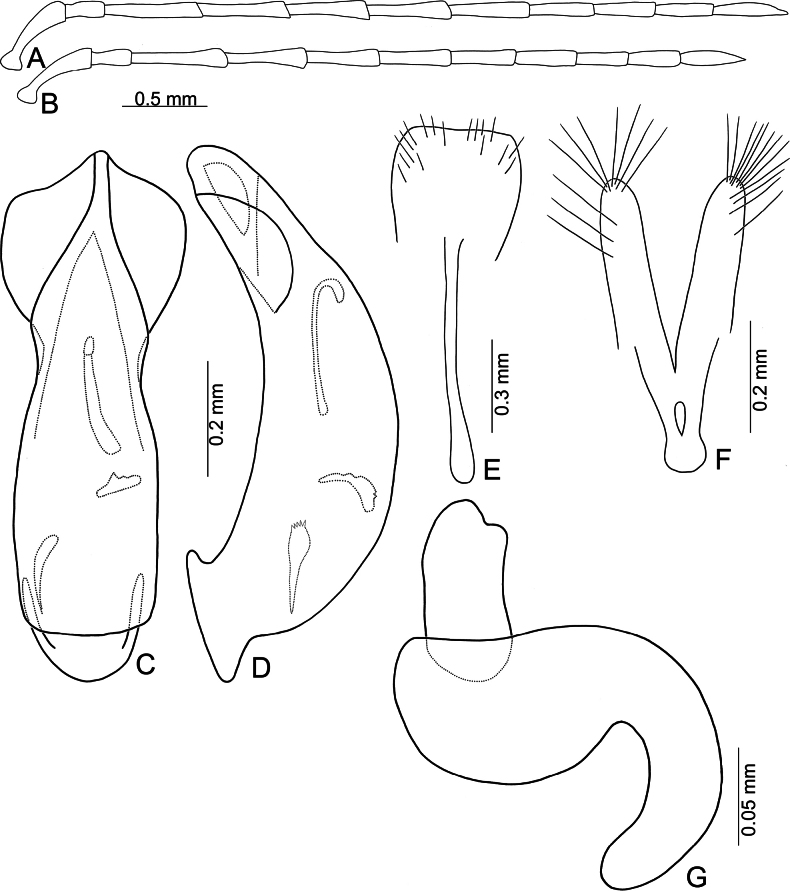
*Anatrimonia albofasciata* (Jacoby), comb. nov. **A**. Antenna, male; **B**. Antenna, female; **C**. Aedeagus, dorsal view; **D**. Aedeagus, lateral view; **E**. Abdominal ventrite VIII, female; **F**. Gonocoxae; **G**. Spermatheca.

#### Additional specimen examined

**(*n* = 6)**. **India** • Meghalaya: 1♂ (JBCB): 3 km E of Tura, 25°30'N, 90°14E, 500–1150 m, 15–22.IV.1999, leg. J. Rolčík; **Laos** • Phongsaly: 1♂, 2♀ (NHMB), Phongsaly env., 21°41–2'N, 102°06–8E, ~1500 m, 28.V. –20.VI.2003, leg. V. Kubáň; **Thailand** • Chiang Mai: 1♀ (NHMB), Ang Khang region, 19°53'45"N, 99°02'45"E, 1600 ± 100 m, 2–7.V.2009, leg. L. Dembický; **China** • Yunnan: 1♀ (TARI), Bulangshan (布朗山), 28.IX.2017, leg. Y.-T. Wang.

#### Diagnosis.

**Figure 6.**
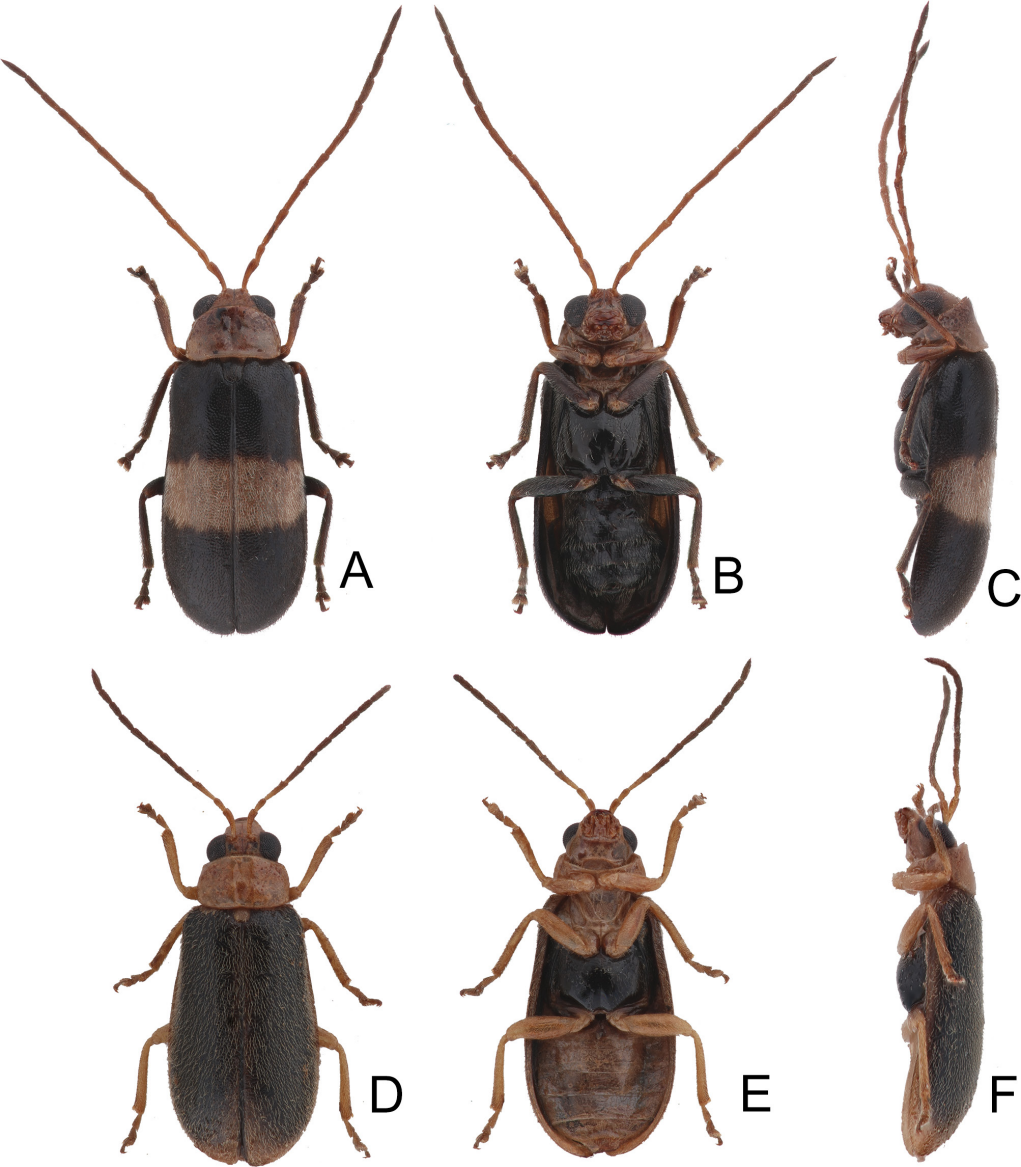
Habitus of *Anatrimonia cheni* sp. nov. and A. chungi sp. nov. **A**. *Anadimonia cheni* sp. nov., male, dorsal view; **B**. Ditto, ventral view; **C**. Ditto, lateral view; **D**. *A. chungi* sp. nov., male, dorsal view; **E**. Ditto, ventral view; **F**. Ditto, lateral view.

**Figure 7.**
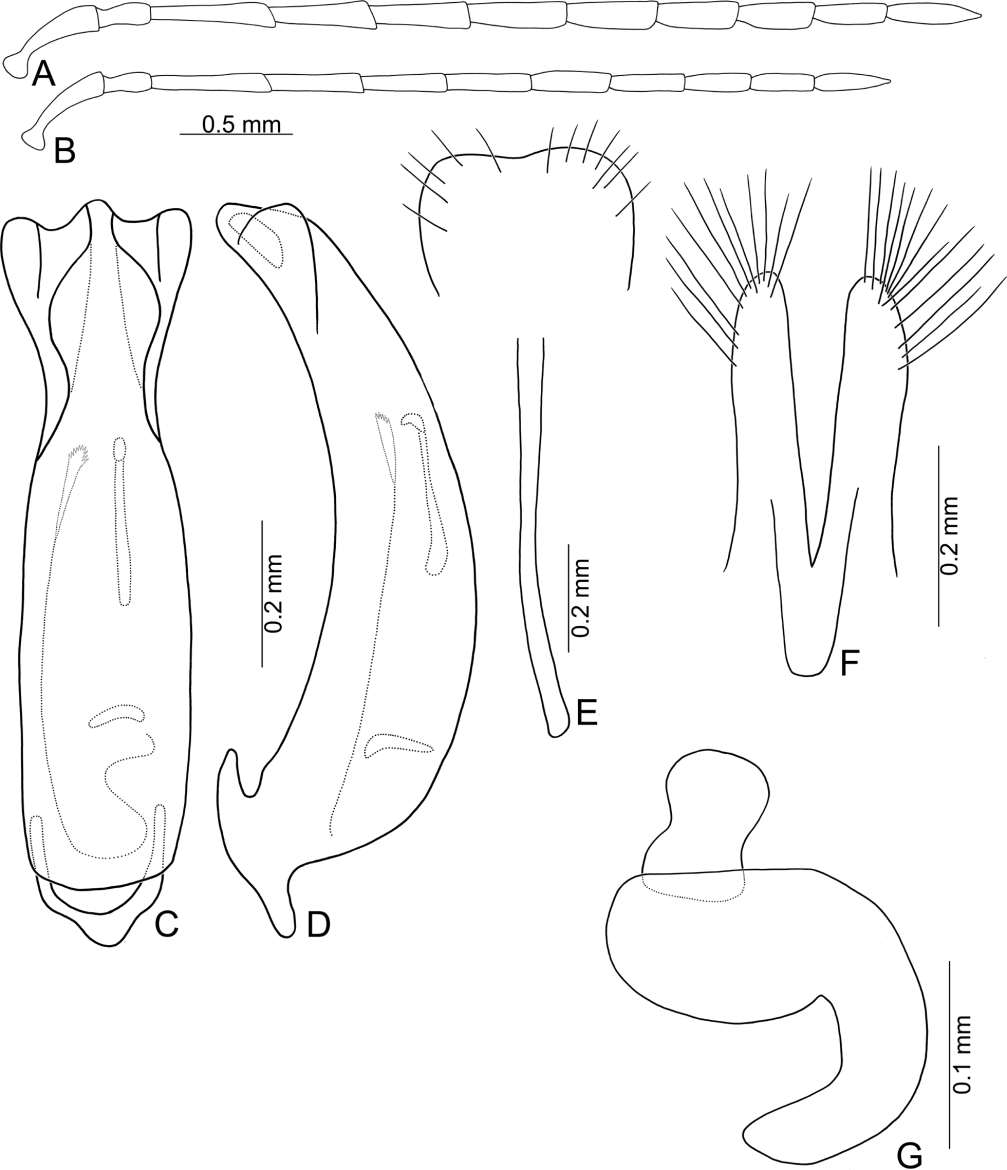
*Anatrimonia cheni* sp. nov. **A**. Antenna, male; **B**. Antenna, female; **C**. Aedeagus, dorsal view; **D**. Aedeagus, lateral view; **E**. Abdominal ventrite VIII, female; **F**. Gonocoxae; **G**. Spermatheca.

Adults of *Anatrimonia albofasciata* (Jacoby, 1892) are similar to those of *A. cheni* sp. nov., *A. hainana* (Gressitt & Kimoto, 1963), and *A. wangi* sp. nov. in possessing yellowish heads and prothoraxes, black elytra with median transverse white bands in both sexes. However, adults of *A. albofasciata* and *A. cheni* sp. nov. have yellow antennae (Figs 4A–C, 6A–C) [black antennae in *A. hainana* and *A. wangi* sp. nov. (Fig. 4D–F)]. Males of *A. albofasciata* lack longitudinal ridges on antennae (with longitudinal ridges on antennomeres IV–X in males of *A. cheni* sp. nov.), bases of dorsal ridges wider than aedeagus (Fig. 5C, D) [bases of dorsal ridges narrower than aedeagus in *A. cheni* sp. nov. (Fig. 7C, D)], endophallic sac composed of three small sclerites (Fig. 5C, D) [one extremely slender, flagellum like endophallic sclerite and two smaller ones in *A. cheni* sp. nov. (Fig. 7C, D)].

#### Description.

Length 4.5–5.0 mm, width 2.0–2.1 mm. General color black (Fig. 4A–C); head and prothorax pale yellow, antenna yellowish-brown; middle and hind legs black but trochanters yellowish-brown, front legs pale yellow, but apical 3/2 of tibia, and tarsi dark brown; each elytron with one transverse white band at middle. Antennae (Fig. 5A) filiform in males, ratio of lengths of antennomeres I–XI 1.0 : 0.5 : 1.1 : 1.0 : 1.0 : 1.0 : 1.0 : 0.8 : 0.7 : 0.7 : 0.9; ratio of length to width of antennomeres I–XI 4.0 : 2.4 : 6.2 : 5.5 : 4.8 : 4.6 : 5.0 : 4.4 : 4.4 : 4.3 : 5.6; shorter in females, ratio of lengths of antennomeres I–XI (Fig. 5B) 1.0 : 0.5 : 1.1 : 1.0 : 0.9 : 0.9 : 0.8 : 0.7 : 0.6 : 0.6 : 0.8; ratio of length to width of antennomeres I–IX 4.2 : 2.6 : 5.0 : 4.0 : 4.0 : 4.0 : 3.8 : 4.0 : 3.4 : 3.4 : 4.5. Pronotum 1.66–1.73 × wider than long; disc with sparse, fine punctures at sides; lateral margins slightly rounded and basally narrowed, basal margin straight, apical broadly rounded; disc with a broad transverse depression at each side, median longitudinal groove obvious. Elytra 1.79–1.84 × longer than wide; with extremely dense setae, parallel sided, apex convergent. Aedeagus (Fig. 5C, E) broad, ~ 3.0 × longer than wide; lateral margins strongly narrowed at apical 2/5, narrower than reverse V-shaped ridge; apical margin truncate, moderately convex at middle; apico-lateral expansion widest and recurved; dorsal disc with reverse V-shaped ridge from near apex to apical 2/5, basally curved; strongly curved in lateral view; endophallic sac with three small sclerites, anterior sclerite slender, middle one transverse and with irregular shape, posterior sclerite tapering basally, with several teeth at apex. Gonocoxae (Fig. 5F) cylindrical, apex widely rounded; each gonocoxa with 10–12 long setae at apex, both gonocoxae basally connected with one longitudinal sclerite, basally widened and rounded, with one membranous area near base. Ventrite VIII (Fig. 5E) weakly sclerotized, apical margin truncate; disc with sparse short setae near apical and apico-lateral margin; spiculum slender and long. Spermathecal receptaculum (Fig. 5G) moderately swollen; pump wide and curved; sclerotized spermathecal duct wide but short.

#### Food plants.

Unknown.

#### Distribution.

China, India, Laos, Myanmar, and Thailand.

### Anatrimonia cheni sp. nov.

Taxon classificationAnimaliaColeopteraChrysomelidae

072A8771-D021-5D36-A1E5-9AE41268481D

https://zoobank.org/A7412506-F6B7-4969-B558-BEE7E61F00D4

Figs 6A–C, 7

#### Type specimens examined

**(*n* = 9). *Holotype*** ♂ (TARI), **China** • **Yunnan**: Banggunjianshan (邦棍尖山), 15.IX.2015, leg. Y.-T. Wang. ***Paratypes***. • 1♂ (TARI), same data as holotype; • 1♀ (TARI), same but with “27.VI.2017”; **China** • Yunnan: 1♀ (TARI), Bulangshan (布朗山), 28.IX.2017, leg. Y.-T. Wang; • 1♀ (TARI), Mohan (磨憨), 14.V.2016, leg. Y.-T. Wang; **Laos** • Houa Phan: 1♂, 1♀ (NMPC), Ban Saluei → Phou Pane Mt., 20°12–13.5'N, 103°59.5'–104.01E, 1340–1870 m, 2–22.VI.2011, leg. V. Kubáň & Lao coll.; • 1♂, 2♀ (NMPC), same locality, 15.IV.–15.V.2008, leg. Lao collectors.

#### Diagnosis.

Adults of *Anatrimonia cheni* sp. nov. are similar to those of *A. albofasciata* (Jacoby, 1892), *A. hainana* (Gressitt & Kimoto, 1963), and *A. wangi* sp. nov. in possessing yellowish heads and prothoraxes, black elytra with median transverse white bands in both sexes. However, adults of *A. cheni* sp. nov. and *A. albofasciata* have yellow antennae (Figs 4A–C, 6A–C) [black antennae in *A. hainana* and *A. wangi* sp. nov. (Fig. 4D–F)]. Adults of *A. cheni* sp. nov. have longitudinal ridges on antennomeres IV–X in males (lacking longitudinal ridges on antenna in males of *A. albofasciata*), base of dorsal ridges narrower than aedeagus (Fig. 7C, D) [base of dorsal ridges wider than aedeagus in *A. albofasciata* (Fig. 5C, D)], endophallic sac composed of one extremely slender, flagellum like sclerite and two small ones (Fig. 7C, D) [three small endophallic sclerites in *A. albofasciata* (Fig. 5C, D)].

#### Description.

Length 5.5–5.9 mm, width 2.2–2.7 mm. General color black (Fig. 6A–C); head and prothorax pale yellow, antenna yellowish-brown; middle and hind legs black but trochanters yellowish-brown, front legs pale yellow, but apical 3/2 of tibia, and tarsi dark brown; each elytron with one transverse white band at middle. Antennae (Fig. 7A) filiform in males, antennomeres IV–X with one longitudinal ridge, ratio of lengths of antennomeres I–XI 1.0 : 0.5 : 1.1 : 0.9 : 0.9 : 0.9 : 0.8 : 0.7 : 0.7 : 0.7 : 0.9; ratio of length to width of antennomeres I–XI 4.1 : 2.4 : 5.0 : 3.6 : 3.4 : 3.4 : 3.2 : 3.0 : 2.9 : 3.4 : 4.6; antennomeres IV–X without longitudinal ridge in females, ratio of lengths of antennomeres I–XI (Fig. 7B) 1.0 : 0.5 : 1.1 : 0.9 : 0.8 : 0.8 : 0.8 : 0.7 : 0.6 : 0.6 : 0.8; ratio of length to width of antennomeres I–XI 4.3 : 2.4 : 5.6 : 5.0 : 4.4 : 4.8 : 4.4 : 4.1 : 3.7 : 3.7 : 4.5. Pronotum 1.65–1.75 × wider than long; disc with sparse, fine punctures at sides; lateral margins slightly rounded and basally narrowed, basal margin straight, apical margin slightly and medially convex; disc with a broad transverse depression at each side, median longitudinal groove obvious. Elytra 1.71–1.88 × longer than wide; with extremely dense setae, parallel sided, apex convergent. Aedeagus (Fig. 7C, D) narrow, ~ 3.8 × longer than wide; lateral margins strongly narrowed at apical 1/4; apical margin concave, moderately convex at middle; apico-lateral expansion widest and forming reverse hook; dorsal disc with reverse V-shaped ridge from near apex to apical 1/4, basally curved; strongly curved in lateral view; endophallic sac with three sclerites, one sclerite slender, another transverse, other sclerite flagellum-like, with several teeth at apex. Gonocoxae (Fig. 7F) cylindrical, apex widely rounded; each gonocoxa with 11–13 long setae at apex, both gonocoxae basally connected with one longitudinal sclerite, base square. Ventrite VIII (Fig. 7E) weakly sclerotized, apical margin truncate, slightly concave at middle; disc with sparse short setae near apical and apico-lateral margin; spiculum slender and long. Spermathecal receptaculum (Fig. 7G) moderately swollen; pump wide and curved; sclerotized spermathecal duct wide but short.

#### Food plants.

Unknown.

#### Etymology.

This new species is named for Mr. Chang-Chin Chen (陳常卿) who assisted the study in various ways.

#### Distribution.

China (Yunnan) and Laos.

### Anatrimonia chungi sp. nov.

Taxon classificationAnimaliaColeopteraChrysomelidae

5905657D-AAFF-5CDC-A570-C02B45F0B22B

https://zoobank.org/ADFC0921-F17B-4B92-9809-288F3A4E0661

Figs 6D–F, 8

#### Type specimens examined

**(*n* = 9). *Holotype*** ♂ (TARI), **Taiwan** • **Kaohsiung**: Tengchih (藤枝), 18.IV.2013, leg. B.-X. Guo. ***Paratypes***. • 1♂, 2♀ (TARI), same but with “leg. Y.-T. Chung”; • **Hualien**: 1♂ (TARI), Hsilin trail (西林林道), 29.VII.2025, leg. J.-C. Chen; • **Kaohsiung**: 1♂ (TARI), Chungchihkuan (中之關), 12.X.2012, leg. L.-P. Hsu; • 1♂, Tengchih trail (藤枝林道), 13.IV.2013, leg. W.-C. Liao; • **Nantou**: 1♂ (TARI), Choshe (卓社), 8.VII.2017, leg. B.-H. Kuo (= B.-X. Guo); • 1♀ (TARI), Huisunlinchang (惠蓀林場), 22.IV.2015, leg. B.-X. Guo; • 1♂ (TARI), Shanlinhsi (杉林溪), 24.VIII.2013, leg. W.-C. Liao; • **Taoyuan**: 1♂ (Taiwan), Fufushan (夫婦山), 5.IV.2015, leg. M.-H. Tsou.

**Figure 8.**
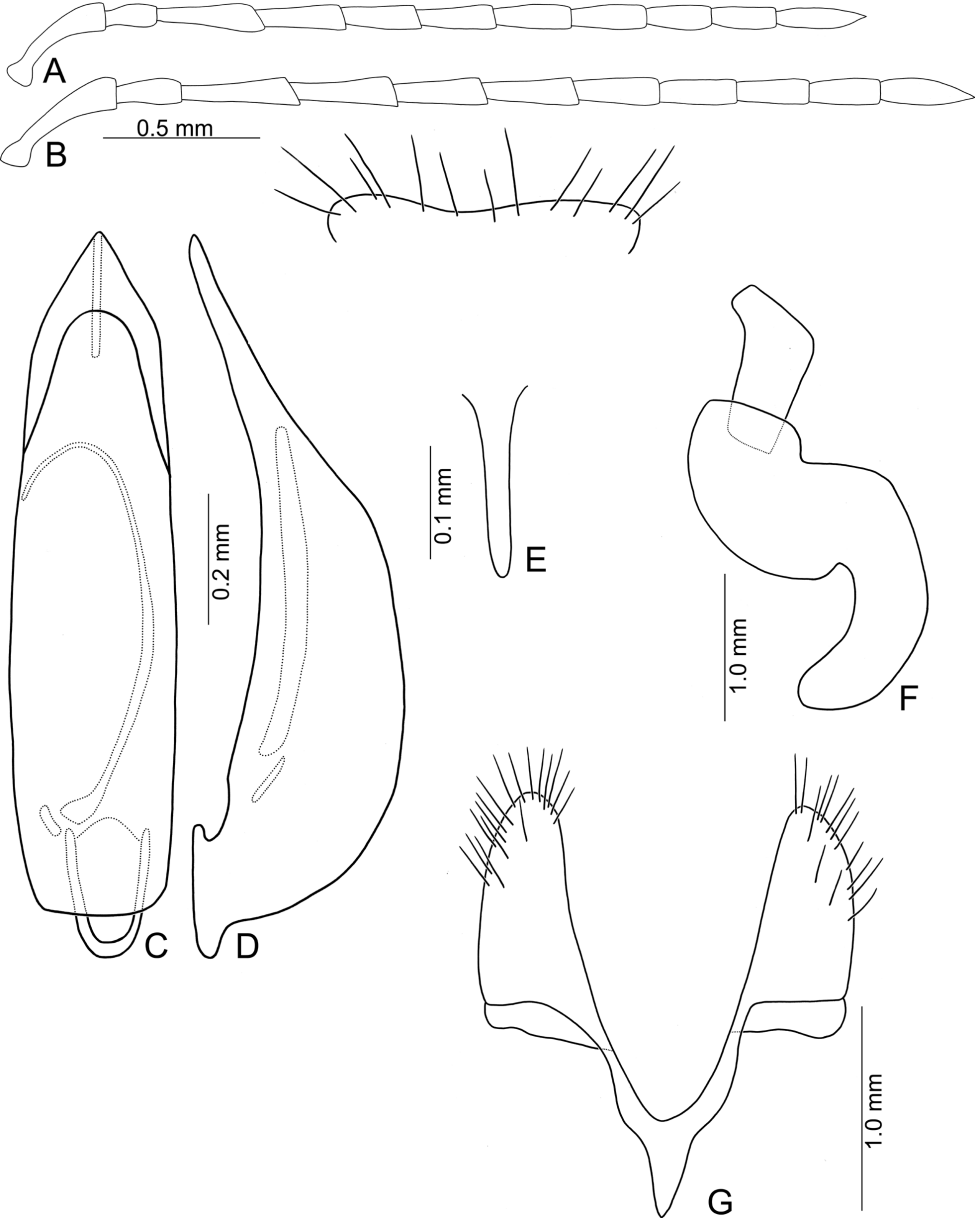
*Anatrimonia chungi* sp. nov. **A**. Antenna, male; **B**. Antenna, female; **C**. Aedeagus, dorsal view; **D**. Aedeagus, lateral view; **E**. Abdominal ventrite VIII, female; **F**. Spermatheca; **G**. Gonocoxae.

#### Diagnosis.

Adults of *Anatrimonia chungi* sp. nov. are similar to those of *A. tsoui* sp. nov. have yellow heads and prothoraxes, and black elytra in males. However, adults of *A. chungi* sp. nov. have black elytra in females (black elytra with median transverse white bands in most females of *A. tsoui* sp. nov.), yellowish antennae, legs, scutellum, and abdomen (Fig. 6D–F) [black middle and hind legs, scutellum, and abdomen in *A. tsoui* sp. nov. (Fig. 16)], aedeagus without apico-lateral expansion and dorsal ridges (Fig. 8C, D) [with apico-lateral expansion and dorsal ridges in *A. tsoui* sp. nov. (Fig. 17C, D)], endophallic sac composed of one extremely slender, flagellum-like sclerite and another small sclerite (Fig. 8C, D) [three small endophallic sclerites in *A. tsoui* sp. nov. (Fig. 17C, D)].

#### Description.

Length 4.0–4.2 mm, width 2.0–2.2 mm. General color black (Fig. 6A–C); head, prothorax, and scutellum pale yellow; antennomeres IV–XI and apex of III brown; tarsi and apical half of tibia darkened; lateral margins and apices of elytra paler in some individuals. Antennae (Fig. 8A) filiform in males, ratio of lengths of antennomeres I–XI 1.0 : 0.4 : 0.9 : 0.7 : 0.6 : 0.6 : 0.6 : 0.6 : 0.5 : 0.5 : 0.8; ratio of length to width of antennomeres I–XI 4.1 : 2.2 : 3.9 : 3.5 : 3.1 : 3.2 : 3.2 : 3.1 : 2.7 : 3.0 : 4.4; similar in females, ratio of lengths of antennomeres I–XI (Fig. 8B) 1.0 : 0.5 : 0.9 : 0.8 : 0.7 : 0.7 : 0.7 : 0.6 : 0.5 : 0.5 : 0.7; ratio of length to width of antennomeres I–XI 4.1 : 2.4 : 4.3 : 3.7 : 3.2 : 3.3 : 3.1 : 3.0 : 2.8 : 2.8 : 3.6. Pronotum 1.75–1.82 × wider than long; disc with dense, coarse and fine punctures, but reduced in median groove; lateral margins slightly rounded and basally narrowed, apical and basal margin straight; disc with a broad transverse depression at each side, median longitudinal groove obvious. Elytra 1.65–1.73 × longer than wide; with extremely dense setae, lateral margins broadly rounded, widest at apical 1/3, apex convergent. Aedeagus (Fig. 8C, D) narrow, ~ 4.5 × longer than wide; parallel-sided, apically tapering from apical 1/7; dorsal disc without ridges apically; slightly curved in lateral view; endophallic sac with two sclerites, one small, other flagellum-like. Gonocoxae (Fig. 8G) cylindrical, apex widely rounded; each gonocoxa with 14–17 short setae at apex, both gonocoxae basally connected with narrow sclerite, connection triangular. Ventrite VIII (Fig. 8E) well sclerotized, apical margin subtruncate; disc with sparse, long setae near apical margin; spiculum slender and short. Spermathecal receptaculum (Fig. 8F) moderately swollen; pump wide and curved; sclerotized spermathecal duct wide but short.

#### Food plants.

Lauraceae: *Camphora officinarum* Boerh. ex Fabr.

#### Etymology.

This new species is named for Yi-Ting Chung (鍾奕霆), the first member of TCRT to collect specimens of this new species.

#### Distribution.

This new species is widespread in Taiwan but is uncommonly collected.

### Anatrimonia hainana

Taxon classificationAnimaliaColeopteraChrysomelidae

(Gressitt & Kimoto, 1963) stat. res. comb. nov.

21A7114F-D364-54CC-B992-8334A1306A4B

Figs 4D–F, 9

Trichocerophysa hainana Gressitt & Kimoto, 1963: 473 (China: Hainan); Kimoto 1989: 33 [as a synonymy of Anadimonia latifascia (Gressitt & Kmoto, 1963)].

#### Type specimen examined.

***Holotype*** • (Fig. 4D–F) ♂ (CAS, by original designation): “Dwa Bi, Hainan Id / VII-28-35 [p, w] // L. Gressitt / Collection [p, w] // L. Gressitt / Collection [p, w] // HOLOTYPE [p] Trichocerophysa / hainana [h] / Gressitt & Kimoto [p, r] // Trichocerophysa / hainana / G & K [h] / Gressitt & Kimoto det. 1961 [p, w] // California Academy / of Sciences / Type / No. [p] 13313 [h, w]”.

#### Additional specimen examined

**(*n* = 2)**. **China** • Hainan: 1♂, 1♀ (TARI), Jianfengling (尖峰嶺), 10.V.2015, leg. Y.-T. Wang.

#### Diagnosis.

**Figure 9.**
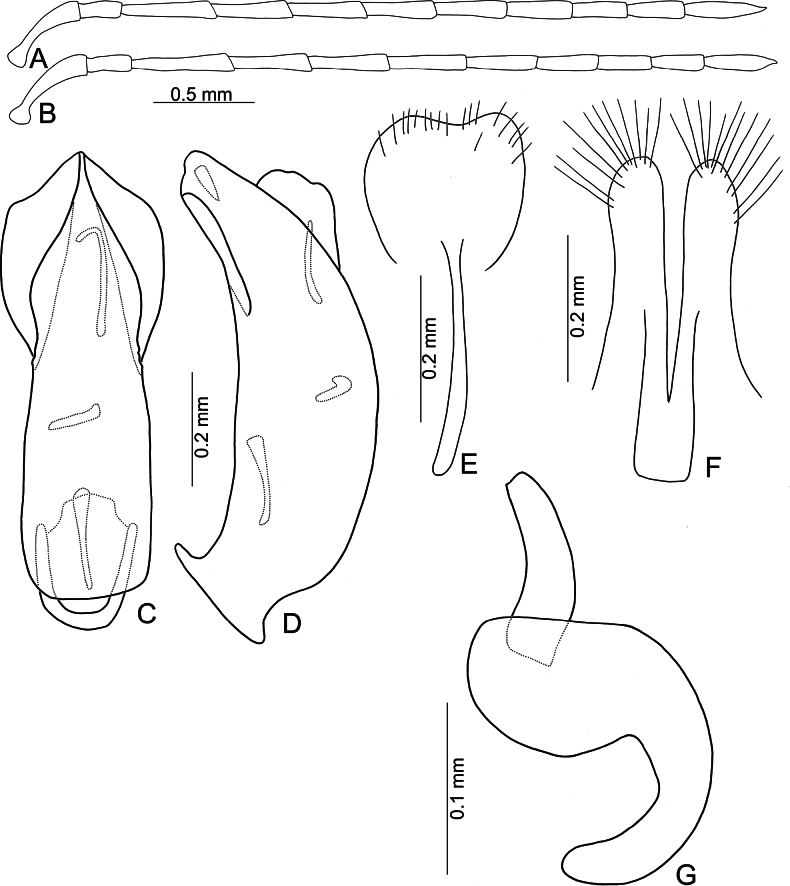
*Anatrimonia hainana* (Gressitt & Kimoto, 1963) stat. rec. & comb. nov. **A**. Antenna, male; **B**. Antenna, female; **C**. Aedeagus, dorsal view; **D**. Aedeagus, lateral view; **E**. Abdominal ventrite VIII, female; **F**. Gonocoxae; **G**. Spermatheca.

Adults of *Anatrimonia hainana* (Gressitt & Kimoto, 1963) are similar to those of *A. albofasciata* (Jacoby, 1892), *A. cheni* sp. nov., and *A. wangi* sp. nov. in possessing yellowish heads and prothoraxes, black elytra with median transverse white bands in both sexes. However, adults of *A. hainana* and *A. wangi* sp. nov. have black antennae (Fig. 4D–F) [yellow antennae in *A. albofasciata* and *A. cheni* sp. nov. (Figs 4A–C, 6 A–C)]. Adults of *A. hainana* have black scutellum and abdomen (yellow scutellum and abdomen in *A. wangi* sp. nov.), aedeagus with dorsal ridge but not projecting anterior from apex of aedeagus (Fig. 9C, D) [aedeagus with dorsal ridge projecting anterior from apex of aedeagus in *A. wangi* sp. nov. (Fig. 18C, D)], endophallic sac composed of three small endophallic sclerites (Fig. 9C, D) [one extremely slender, flagellum like endophallic sclerite and three small ones in *A. wangi* sp. nov. (Fig. 18C, D)].

#### Description.

Length 4.1–5.2 mm, width 1.7–2.3 mm. General color black; head and prothorax pale yellow, antenna blackish-brown; middle and hind legs black but trochanters yellowish-brown, front legs pale yellow, but apical 3/2 of tibia, and tarsi dark brown; each elytron with one transverse white band at middle. Antennae (Fig. 9A) filiform in males, ratio of lengths of antennomeres I–XI 1.0 : 0.5 : 1.0 : 0.9 : 0.8 : 0.8 : 0.8 : 0.7 : 0.6 : 0.6 : 0.9; ratio of length to width of antennomeres I–XI 4.5 : 2.5 : 5.5 : 4.5 : 4.3 : 4.2 : 4.2 : 3.7 : 3.3 : 3.3 : 5.3; similar in females, ratio of lengths of antennomeres I–XI (Fig. 9B) 1.0 : 0.5 : 1.0 : 0.8 : 0.8 : 0.8 : 0.7 : 0.6 : 0.5 : 0.5 : 0.8; ratio of length to width of antennomeres I–XI 4.5 : 2.5 : 5.5 : 4.6 : 4.5 : 4.9 : 4.4 : 3.9 : 3.4 : 3.3 : 4.6. Pronotum 1.71–1.74 × wider than long; disc with sparse, fine punctures at sides; lateral margins slightly rounded and basally narrowed, basal margin straight, apical margin slightly and medially convex; disc with a broad transverse depression at each side, median longitudinal groove obvious. Elytra 1.70–1.92 × longer than wide; with extremely dense setae, parallel sided, apex convergent. Aedeagus (Fig. 9C, D) broad, ~ 3.0 × longer than wide; lateral margins strongly narrowed at apical 2/5; slightly and subapically narrowed; apico-lateral margins widest and recurved; dorsal disc with reverse V-shaped ridge from near apex to apical 2/5, irregular near base; strongly curved in lateral view; endophallic sac with three sclerites, two sclerites slender, other sclerite transverse. Gonocoxae (Fig. 9F) cylindrical, apex widely rounded; each gonocoxa with 11 or 12 long setae at apex, both gonocoxae basally connected with one longitudinal sclerite, base square. Ventrite VIII (Fig. 9E) weakly sclerotized, apical margin concave at middle; disc with sparse short setae near apical and apico-lateral margin; spiculum slender and long. Spermathecal receptaculum (Fig. 9G) moderately swollen; pump wide and curved; sclerotized spermathecal duct wide but short.

#### Variation.

The holotype of *Trichocerophysa hainana* possesses entirely black elytra (Fig. 4D).

#### Food plants.

Unknown.

#### Distribution.

China (Hainan)

### Anatrimonia huangi sp. nov.

Taxon classificationAnimaliaColeopteraChrysomelidae

0A19ABD2-9521-58EB-A52B-922C4A2FC4B8

https://zoobank.org/3E97BB63-2E20-4E00-BAED-5F4FFE68F500

Figs 10A–F, 11

#### Type specimens examined

**(*n* = 11). *Holotype*** ♂ (TARI), **Taiwan** • **Nantou**: Peitungyanshan (北東眼山), 3.VII.2014, leg. F.-S. Huang, 變葉新木薑子 (*Neolitsea variabillima*), 噴霧 (fogging). ***Paratypes***. • 5♂, 1♀ (TARI), same data as holotype; • 2♂, 4♀ (TARI), same locality and collector, 16.IX.2013; • **Taichung**: 2♂, 1♀ (TARI), Fushoushan (福壽山), 18.VII.2021, leg. J.-C. Chen; • 1♀ (TARI), same but with “20.VII.2021”.

#### Diagnosis.

**Figure 10.**
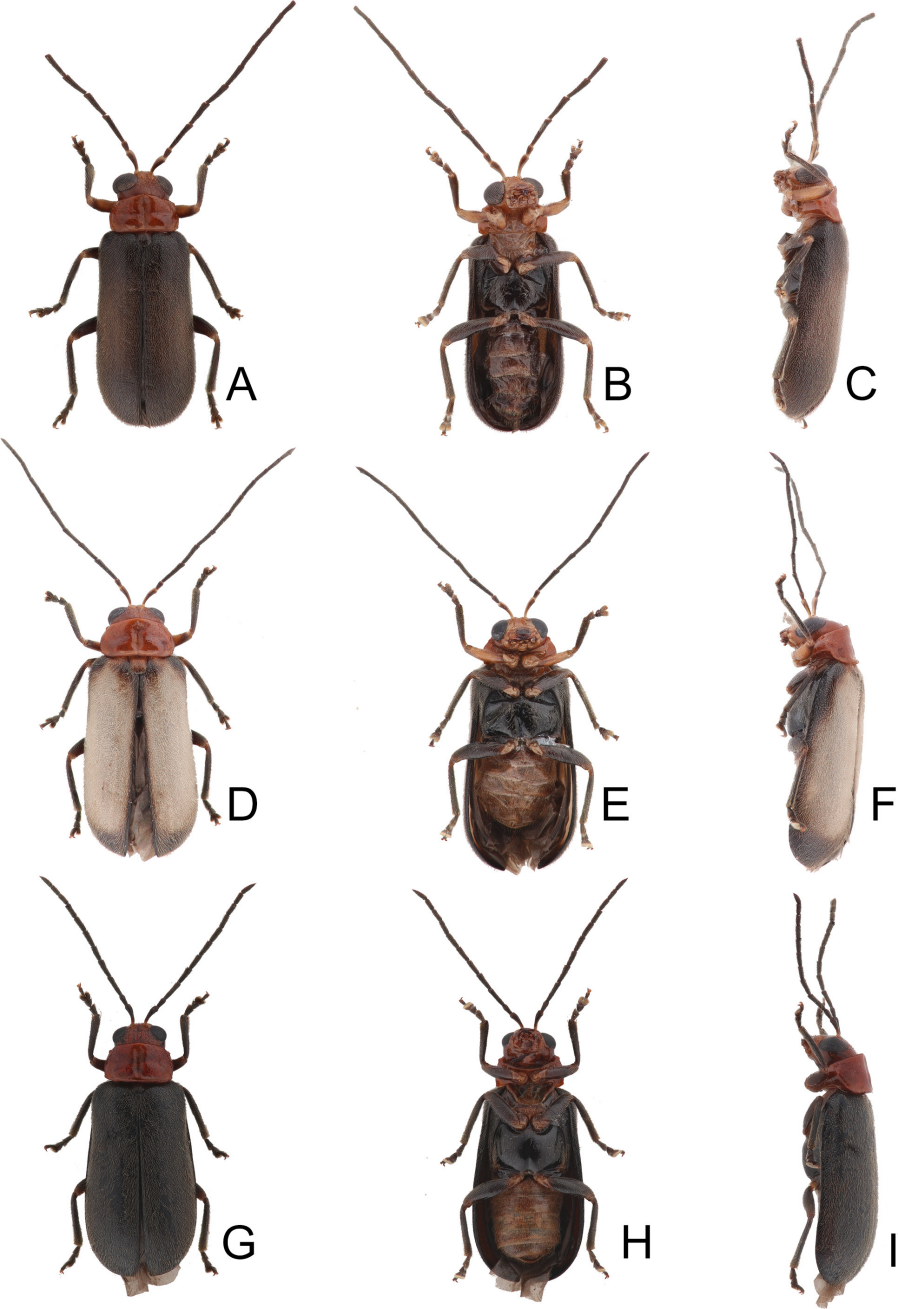
Habitus of *Anatrimonia huangi* sp. nov. and *A. meihuai* sp. nov. **A**. *Anadimonia huangi* sp. nov., male, dorsal view; **B**. Ditto, ventral view; **C**. Ditto, lateral view; **D**. *A. huangi* sp. nov., female, dorsal view; **E**. Ditto, ventral view; **F**. Ditto, lateral view; **G**. *A. meihuai* sp. nov., female, dorsal view; **H**. Ditto, ventral view; **I**. Ditto, lateral view.

**Figure 11.**
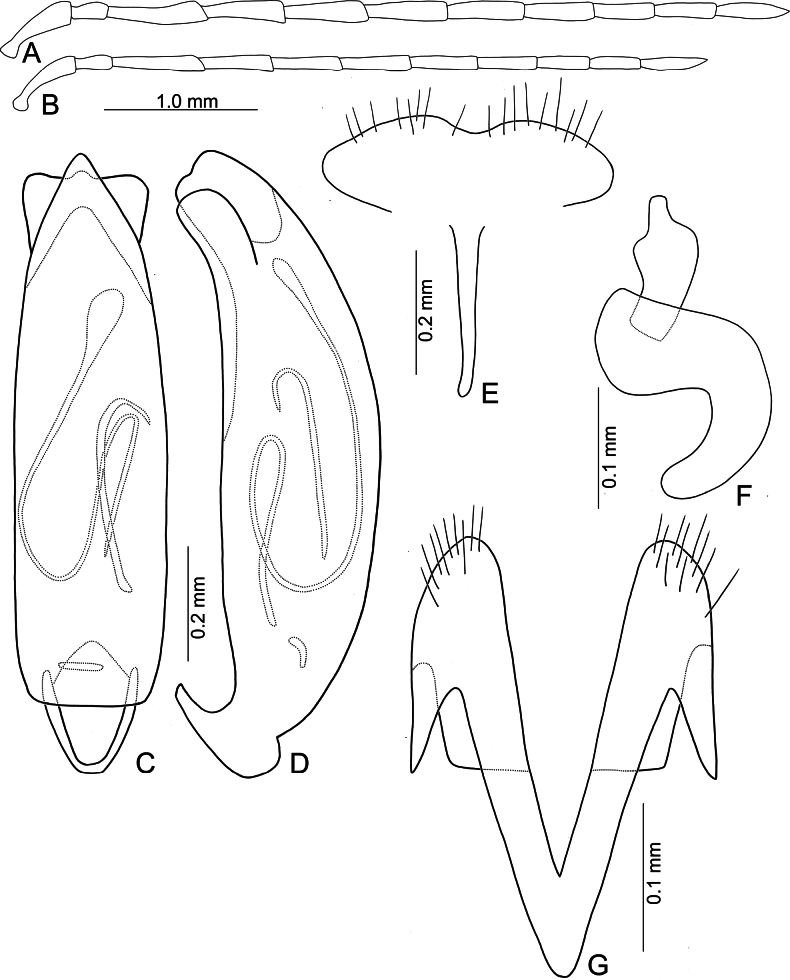
*Anatrimonia huangi* sp. nov. **A**. Antenna, male; **B**. Antenna, female; **C**. Aedeagus, dorsal view; **D**. Aedeagus, lateral view; **E**. Abdominal ventrite VIII, female; **F**. Spermatheca; **G**. Gonocoxae.

Adults of *Anatrimonia huangi* sp. nov. and *A. meihuai* sp. nov. can be separated from other species by the black elytra and reddish-brown head and prothorax (Fig. 10, 15A–C). However, adults of *A. huangi* sp. nov. possess a median transverse white band on the elytra (Fig. 10A–F) [without median transverse white band on the elytra in *A meihuai* sp. nov. (Fig. 10G–I)], apically tapering dorsal ridges projecting from apex of aedeagus (Fig. 11C, D) [widely rounded apex of dorsal ridges not projecting from apex of aedeagus in *A. meihuai* sp. nov. (Fig. 14C, D)], endophallic sac composed of one extremely slender, flagellum-like sclerite, another slender sclerite, and the other small sclerite (Fig. 11C, D) [three small endophallic sclerites in *A. meihuai* sp. nov. (Fig. 14C, D)].

#### Description.

Length 4.6–5.7 mm, width 1.9–2.3 mm. General color black (Fig. 10A–F); head and prothorax reddish-yellow, antenna blackish-brown but two basal antennomeres paler; middle and hind legs black, front legs reddish-yellow, but apical 2/3 of tibia, and tarsi dark brown; scutellum reddish-brown, elytra grayish-white except for base and apex in females, but more or less reduced in males. Antennae (Fig. 11A) filiform in males, ratio of lengths of antennomeres I–XI 1.0 : 0.5 : 1.2 : 1.0 : 1.0 : 1.0 : 1.0 : 0.9 : 0.8 : 0.8 : 0.9; ratio of length to width of antennomeres I–XI 3.4 : 2.2 : 4.6 : 3.8 : 3.8 : 4.3 : 4.3 : 3.9 : 3.8 : 4.0 : 4.8; more slender and shorter in females, ratio of lengths of antennomeres I–XI (Fig. 11B) 1.0 : 0.5 : 1.2 : 1.0 : 0.9 : 0.9 : 0.9 : 0.8 : 0.7 : 0.7 : 0.9; ratio of length to width of antennomeres I–XI 4.2 : 2.5 : 5.6 : 4.5 : 4.8 : 4.8 : 4.9 : 4.3 : 3.8 : 4.0 : 5.3. Pronotum 1.89–1.95 × wider than long; disc with dense, coarse punctures at sides; lateral margins slightly rounded and basally narrowed, basal margin straight, apical margin straight but convex at middle; disc with a broad transverse depression at each side, median longitudinal groove obvious. Elytra 1.89–1.90 × longer than wide; with extremely dense setae, parallel sided, apex convergent. Aedeagus (Fig. 11C, D) broad, ~ 4.0 × longer than wide; lateral margins narrowed at apical 1/5, apical margin concave, slightly convex at middle; apico-lateral margins recurved; dorsal disc with reverse V-shaped ridge apically, apex projecting from apical margin of aedeagus; slightly curved in lateral view; endophallic sac with three sclerites, one small near base of aedeagus, another flagellum-like, the other slender. Gonocoxae (Fig. 11G) cylindrical, apex widely rounded; each gonocoxa with 9–11 short setae at apex, both gonocoxae basally connected with narrow sclerite, connection triangular. Ventrite VIII (Fig. 11E) well sclerotized, apical margin rounded but concave at middle; disc with sparse short setae near apical margin; spiculum slender and short. Spermathecal receptaculum (Fig. 11F) moderately swollen; pump wide and curved; sclerotized spermathecal duct wide but short.

#### Variation.

Populations from Fushoushan (福壽山) have black elytra and scutella (grayish-white bands on elytra reduced).

#### Food plants.

Lauraceae: *Neolitsea variabillima* (Hayata) Kaneh. & Sasaki.

#### Etymology.

This new species is named for Fu-Sheng Huang (黃福盛), the first person to collect specimens of this new species.

#### Distribution.

Central Taiwan.

### Anatrimonia jungchani sp. nov.

Taxon classificationAnimaliaColeopteraChrysomelidae

64944F20-5D6F-5F0E-8391-BBA67C8A9E05

https://zoobank.org/C7A01565-5A3C-4A1A-8C0E-44AA467ACEAF

Figs 12A–C, 13

#### Type specimens examined

**(*n* = 13). *Holotype*** ♂ (TARI), **Taiwan** • **Taitung**: Hsiangyang (向陽), 5.VII.2024, leg. J.-C. Chen. ***Paratypes***. • 5♂, 2♀ (TARI), same data as holotype; • **Kaohsiung**: 1♂ (TARI), Kuanshanyakou (關山啞口), 30.VII.2015, leg. C.-F. Lee; • **Pingtung**: 1♀ (TARI), Peitawushan (北大武山), 25.VI.2012, leg. J.-C. Chen; • 1♀ (TARI), same but with “23.VII.2013”; • 1♂, 1♀ (TARI), same but with “14.VI.2022”; • 1♂ (TARI), Tahanshan (大漢山), 29.VI.2018, leg. Y.-T. Chung.

#### Diagnosis.

**Figure 12.**
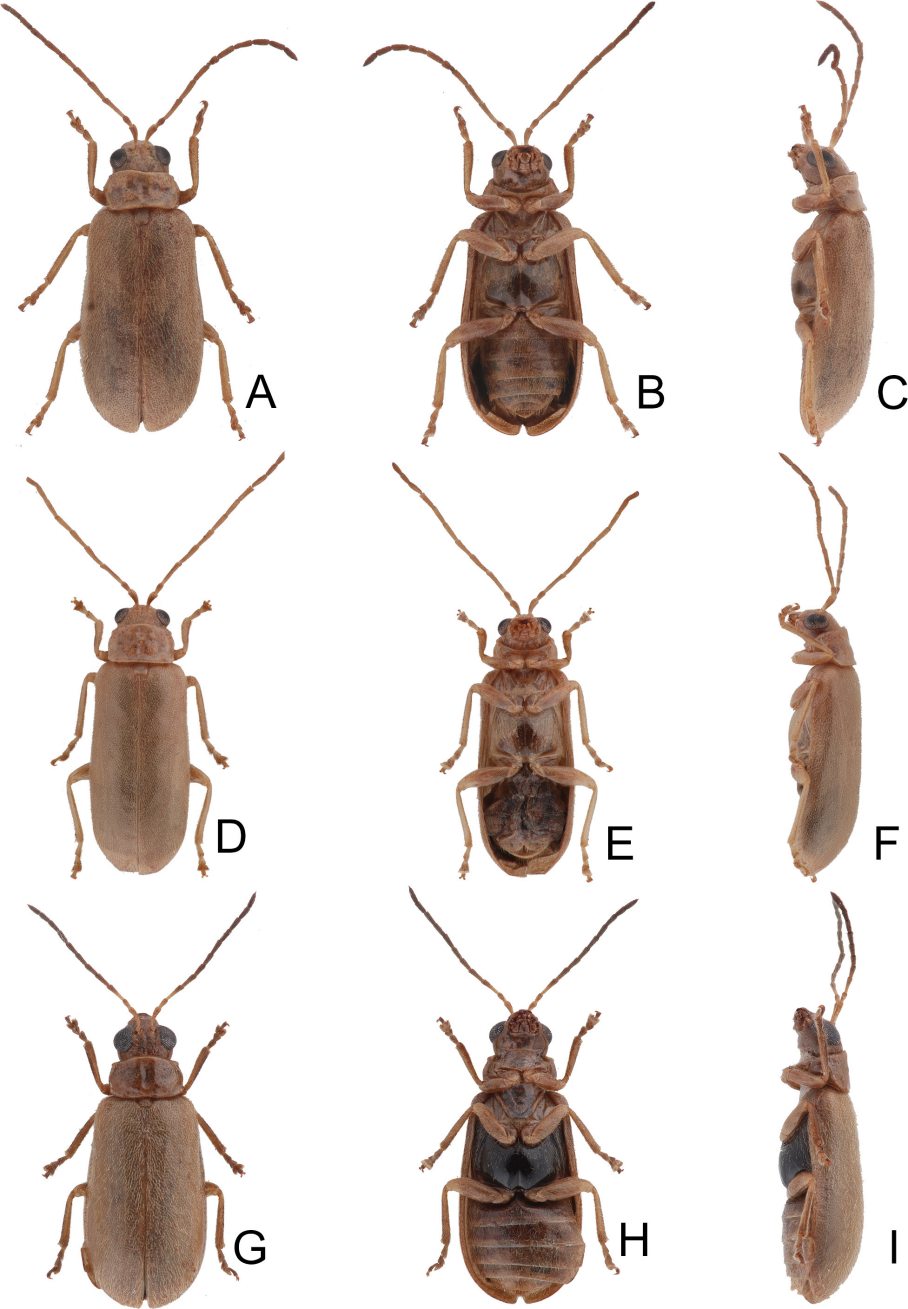
Habitus of *Anatrimonia jungchani* sp. nov., *A. yuae* sp. nov., and *A. yunnanensis* sp. nov. **A**. *Anadimonia jungchani* sp. nov., female, dorsal view; **B**. Ditto, ventral view; **C**. Ditto, lateral view; **D**. *A. yuae* sp. nov., male, dorsal view; **E**. Ditto, ventral view; **F**. Ditto, lateral view; **G**. *A. yunnanensis* sp. nov., female, dorsal view; **H**. Ditto, ventral view; **I**. Ditto, lateral view.

Adults of *Anatrimonia jungchani* sp. nov., *A. yuae* sp. nov., and *A. yunnanensis* sp. nov. are characterized their yellow elytra (Fig. 12). Adults of *A. jungchani* sp. nov. are similar to those of *A. yuae* sp. nov. with large body sizes (> 4.3 mm) and yellow metathoracic ventrites (Fig. 12A–F) [small body sizes (< 4.3 mm) and black metathoracic ventrites in *A. yunnanensis* sp. nov. (Fig. 12G–I)]. The aedeagus of these species are diagnostic: gradually and apically narrowed but narrowly rounded apex in *A. jungchani* sp. nov. (Fig. 11C) [strongly and apically narrowed but with acute apex in *A. yuae* sp. nov. (Fig. 19C), and strongly and apically narrowed but with truncate apex in *A. yunnanensis* sp. nov. (Fig. 20C)]; endophallic sac composed of one slender endophallic sclerites and two small sclerites in *A. jungchani* sp. nov. and *A. yunnanensis* sp. nov. (Figs 11A, 11B, 20A, 20B) [only one slender endophallic sclerite in *A. yuae* sp. nov. (Fig. 19A, B)].

#### Description.

**Figure 13.**
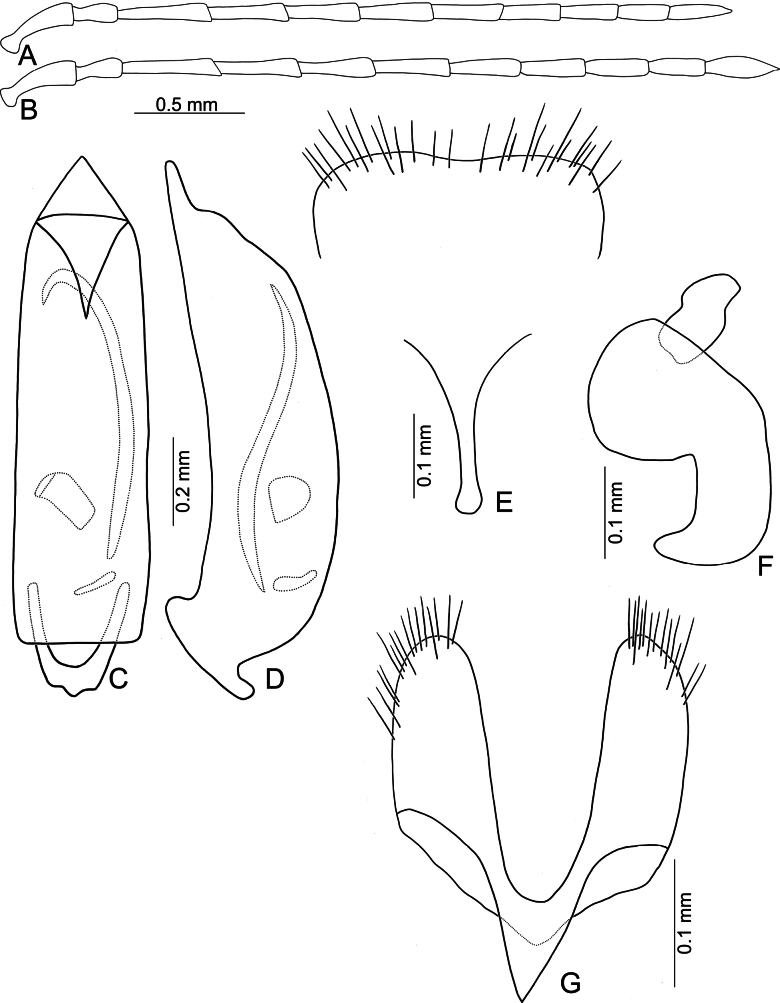
*Anatrimonia jungchani* sp. nov. **A**. Antenna, male; **B**. Antenna, female; **C**. Aedeagus, dorsal view; **D**. Aedeagus, lateral view; **E**. Abdominal ventrite VIII, female; **F**. Spermatheca; **G**. Gonocoxae.

Length 4.4–5.1 mm, width 1.7–2.1 mm. General color pale yellow (Fig. 12A–C); metasternum and abdomen dark brown. Antennae (Fig. 13A) filiform in males, ratio of lengths of antennomeres I–XI 1.0 : 0.6 : 1.1 : 1.0 : 0.9 : 0.9 : 0.9 : 0.7 : 0.7 : 0.6 : 0.8; ratio of length to width of antennomeres I–XI 3.6 : 2.5 : 4.9 : 4.2 : 4.0 : 4.2 : 4.1 : 3.7 : 3.8 : 3.3 : 4.4; similar in females, ratio of lengths of antennomeres I–XI (Fig. 13B) 1.0 : 0.6 : 1.2 : 1.1 : 1.0 : 1.0 : 0.9 : 0.8 : 0.8 : 0.7 : 0.9; ratio of length to width of antennomere I–XI 3.1 : 2.3 : 5.6 : 5.0 : 3.9 : 4.3 : 4.0 : 3.6 : 3.6 : 3.2 : 3.4. Pronotum 1.47–1.56 × wider than long; disc with sparse, fine punctures at sides; lateral margins slightly rounded and basally narrowed, some × straight, apical and basal margins straight; disc with a broad transverse depression at each side, median longitudinal groove obvious. Elytra 1.88–1.94 × longer than wide; with extremely dense setae, parallel sided, apex convergent. Aedeagus (Fig. 13C, D) narrow, ~ 4.1 × longer than wide; parallel-sided, apically tapering from apical 1/8; dorsal disc without ridges apically; slightly curved in lateral view; endophallic sac with three sclerites, one simple, small, another extremely slender, others small and dorso-ventrally flattened. Gonocoxae (Fig. 13G) cylindrical, apex widely rounded; each gonocoxa with 15 or 16 short setae at apex, both gonocoxae basally connected with narrow sclerite, connection triangular. Ventrite VIII (Fig. 13E) well sclerotized, apical margin subtruncate, slightly depressed at middle; disc with sparse, long setae near apical margin; spiculum slender and short. Spermathecal receptaculum (Fig. 13F) moderately swollen; pump wide and curved; sclerotized spermathecal duct wide but short.

#### Food plants.

Lauraceae: *Litsea morrisonensis* Hayata, *Neolitsea acuminatissima* Hayata, Kanehira & Sasaki.

#### Etymology.

Etymology. This new species is named for Jung-Chan Chen (陳榮章), the first member of TCRT to collect specimens.

#### Distribution.

South Taiwan.

### Anatrimonia meihuai sp. nov.

Taxon classificationAnimaliaColeopteraChrysomelidae

D70F198A-0A48-5D30-BD70-C1E692DBB321

https://zoobank.org/FDB6B81A-1A39-4F73-B1D2-1AE6A2FDA4C4

**Figure 14.**
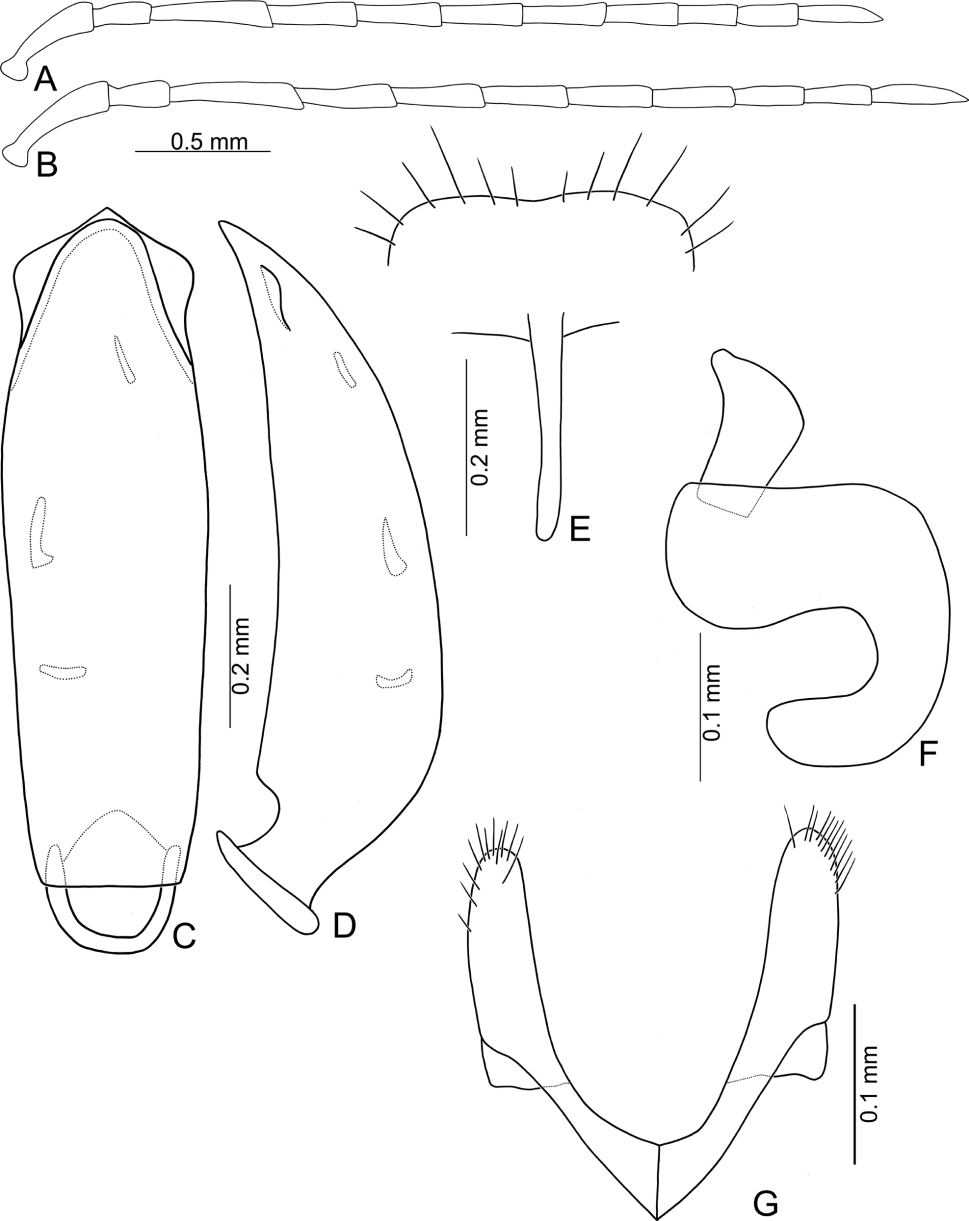
*Anatrimonia meihuai* sp. nov. **A**. Antenna, male; **B**. Antenna, female; **C**. Aedeagus, dorsal view; **D**. Aedeagus, lateral view; **E**. Abdominal ventrite VIII, female; **F**. Spermatheca; **G**. Gonocoxae.

**Figure 15.**
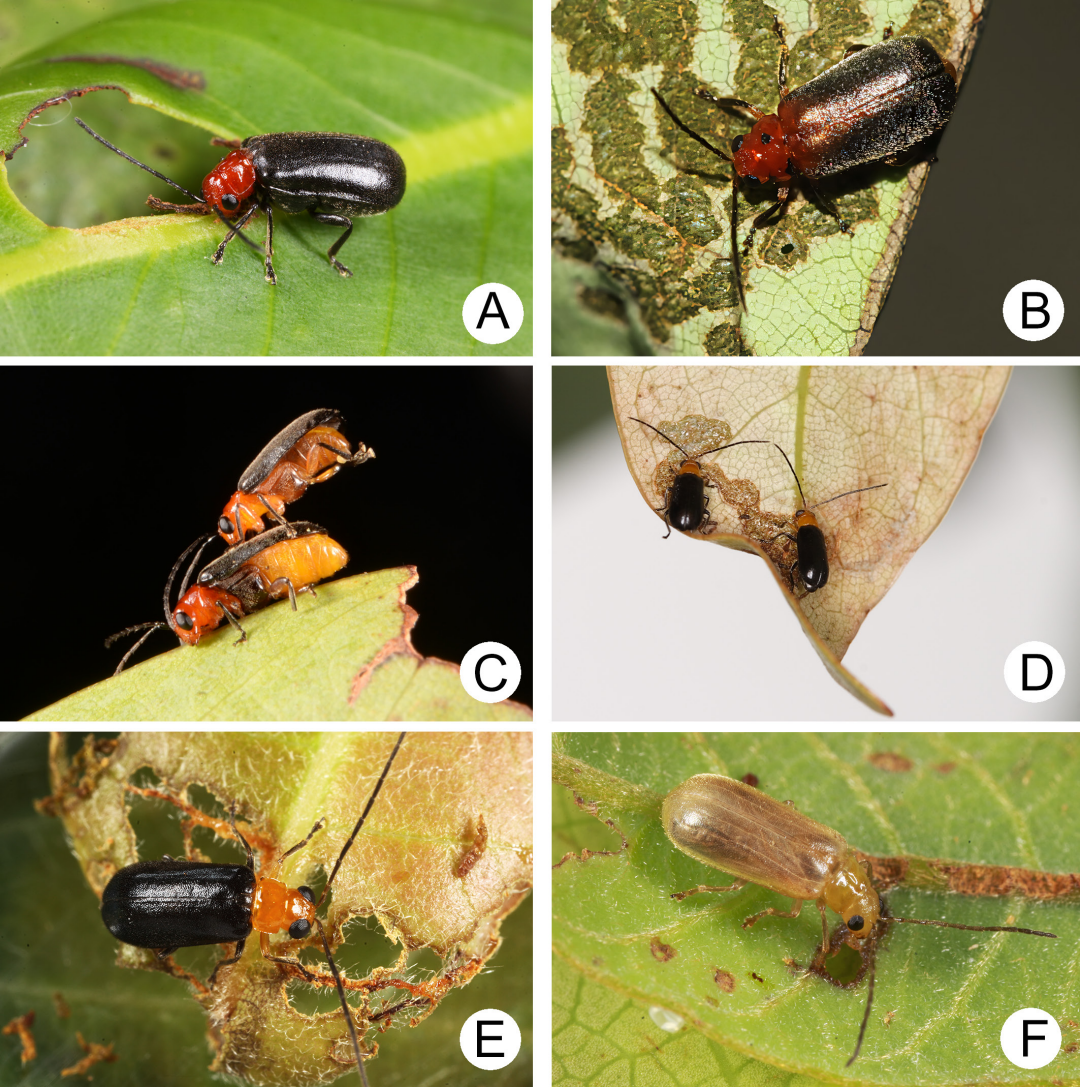
Field photographs of *Anatrimonia* species **A**. Adult of *A. meihuai* sp. nov. feeding on leaf of *Machilus thunbergii*; **B**. Adult of *A. meihuai* sp. nov. feeding on leaf of *Camphora officinarum*; **C**. A couple of Adults of *A. meihuai* sp. nov. resting on leaf of *Machilus thunbergii*; **D**. Adults of *A. tsoui* sp. nov. feeding on underside of leaf of *Machilus thunbergii*; **E**. Adult of *A. tsoui* sp. nov. feeding on leaf of *Litsea elongata* var. *mushaensis* (Hayata) J.C. Liao; **F**. Adult of *A. yuae* sp. nov. feeding on underside of leaf of *Litsea hypophaea* Hayata

Figs 10G–I, 14, 15A–C

#### Type specimens examined

**(*n* = 41). *Holotype*** ♂ (TARI), **Taiwan** • **Pingtung**: Peitawushan (北大武山), 19.VIII.2013, leg. Y.-T. Chung. ***Paratypes***. • 2♂, (TARI), same data as holotype; • 1♂, 2♀ (TARI), same but with “11.VIII.2013”; • 1♂, 1♀ (TARI), same but with “15.VIII.2013”; • 1♂ (TARI), same but with “21.III.2015”; • 1♀ (TARI), same but with “26.V.2017”; • 2♂ (TARI), same but with “23.VII.2025”; • 2♂, 6♀ (TARI), same but with “24.VII.2025”; • 2♀ (TARI), same locality, 23.VII.2013, leg. J.-C. Chen; • 1♀ (TARI), same but with “12.VIII.2025”; • Kaohsiung: 1♀ (TARI), Chuyunshan logging trail (出雲山林道), 24.III.2009, leg. C.-F. Lee; • 1♀ (TARI), Shihshan logging trail (石山林道), 24.III.2009, leg. M.-H. Tsou; • **Chiayi**: 1♀ (TARI), Shihcho (石卓), 20.III.2015, leg. B.-X. Guo; • **Kaohsiung**: 2♀ (TARI), Kuanshanyakou (關山啞口), 30.VII.2015, leg. C.-F. Lee; • **Nantou**: 2♂ (TARI), Huisunlinchang (惠蓀林場), 6.III.2014, leg. F.-S. Huang, 香楠 (*Machilus zuihoensis*), 噴霧 (fogging); • 2♂ (TARI), same but with “3.IV.2014”; • 1♀ (TARI), same but with “12.VI.2014”; • **Pingtung**: 1♂ (TARI), Jinshuiying (浸水營), 21.IV.2011, leg. J.-C. Chen; • 1♂ (TARI), Lilungshan (里龍山), 19.III.2013, leg. J.-C. Chen; • 1♂ (TARI), Machia (瑪家), 17.III.2017, leg. Y.-T. Chung; • 1♂ (TARI), Shouka (壽卡), 2.II.2022, leg. Y.-C. Chiu; • 1♀ (TARI), Tahanshan (大漢山), 28.VI.2015, leg. W.-C. Liao; • 1♀ (TARI), same locality, 4.IV.2017, leg. Y.-T. Chung; • 1♂ (TARI), same but with “23.VII.2020”; • 2♀ (TARI), Taiwu (泰武), 19.VII.2013, leg. J.-C. Chen; • 1♀ (TARI), Tsaopu (草埔), 17.II.2024, leg. J.-C. Chen; • **Taoyuan**: 1♀ (TARI), Paling (巴陵), 30.III.2013, leg. M.-H. Tsou.

#### Diagnosis.

Adults of *Anatrimonia meihuai* sp. nov. and *A. huangi* sp. nov. can be separated from other species by the black elytra, and reddish-brown head and prothorax (Fig. 10). However, adults of *A. meihuai* sp. nov. lack a median transverse white elytral band (Fig. 10G–I) [with more or less median transverse white band on the elytra in *A huangi* sp. nov. (Fig. 10A–F)], widely rounded apex of dorsal ridges not projecting from apex of aedeagus (Fig. 14C, D) [apically tapering dorsal ridges projecting from apex of aedeagus in *A. huangi* sp. nov. (Fig. 11C, D)], endophallic sac composed of three small sclerites (Fig. 14C, D) [one extremely slender, flagellum-like endophallic sclerite, another slender sclerite, and the other small sclerite in *A. huangi* sp. nov. (Fig. 11C, D)].

#### Description.

Length 4.4–5.4 mm, width 1.9–2.4 mm. General color black (Fig. 10G–I); head, prothorax, and scutellum reddish-yellow; antennae and all legs black. Antennae (Fig. 14A) filiform in males, ratio of lengths of antennomeres I–XI 1.0 : 0.5 : 1.1 : 0.8 : 0.8 : 0.8 : 0.8 : 0.7 : 0.6 : 0.6 : 0.8; ratio of length to width of antennomeres I–XI 3.6 : 2.6 : 5.0 : 3.8 : 3.6 : 3.6 : 3.6 : 3.3 : 2.8 : 2.6 : 5.0; similar in females, ratio of lengths of antennomeres I–XI (Fig. 14B) 1.0 : 0.5 : 1.1 : 0.8 : 0.7 : 0.7 : 0.7 : 0.7 : 0.6 : 0.6 : 0.8; ratio of length to width of antennomeres I–XI 3.9 : 2.3 : 5.2 : 4.2 : 3.8 : 3.9 : 3.8 : 3.8 : 3.4 : 3.3 : 5.1. Pronotum 1.72–1.78 × wider than long; disc with dense, coarse punctures, but reduced in median groove; lateral margins slightly rounded and basally narrowed, apical and basal margin straight; disc with a broad transverse depression at each side, median longitudinal groove obvious. Elytra 1.79–1.92 × longer than wide; with extremely dense setae, parallel sided, apex convergent. Aedeagus (Fig. 14C, D) broad, ~ 3.8 × longer than wide; lateral margins narrowed at apical 1/7, apex slightly tapering; apico-lateral margins slightly recurved; dorsal disc with rounded ridge apically; slightly curved in lateral view; endophallic sac with three small sclerites. Gonocoxae (Fig. 14G) cylindrical, apex widely rounded; each gonocoxa with 11–16 short setae at apex, both gonocoxae basally connected with narrow sclerite, connection triangular. Ventrite VIII (Fig. 14E) well sclerotized, apical margin rounded but slightly concave at middle; disc with sparse, long setae near apical margin; spiculum slender and short. Spermathecal receptaculum (Fig. 14F) moderately swollen; pump wide and curved; sclerotized spermathecal duct wide but short.

#### Variations.

Some individuals have reddish-brown bases of the elytra (Fig. 15B) and abdomens. Some have reddish meso- and metathoracic ventrites, and middle and hind femora (Fig. 15C).

#### Biological notes.

Some adults were collected with light traps.

#### Food plants.

Lauraceae: *Camphora officinarum* Boerh. ex Fabr. (Fig. 15B), *Machilus obovatifolius* (Hayata) Kaneh. & Sasaki, *M. thunbergii* Siebold & Zucc. (Fig. 15A), *M. zuihoensis* Hayata.

#### Etymology.

This new species is named for Mei-Hua Tsou (曹美華), the first member of TCRT to collect specimens of this new species.

#### Distribution.

This new species is widespread in Taiwan.

### Anatrimonia tsoui sp. nov.

Taxon classificationAnimaliaColeopteraChrysomelidae

7BCCDECE-BD4C-5E61-A660-3CF1E6B45AE4

https://zoobank.org/36176A77-0097-40F2-AC39-D921935006A6

**Figure 16.**
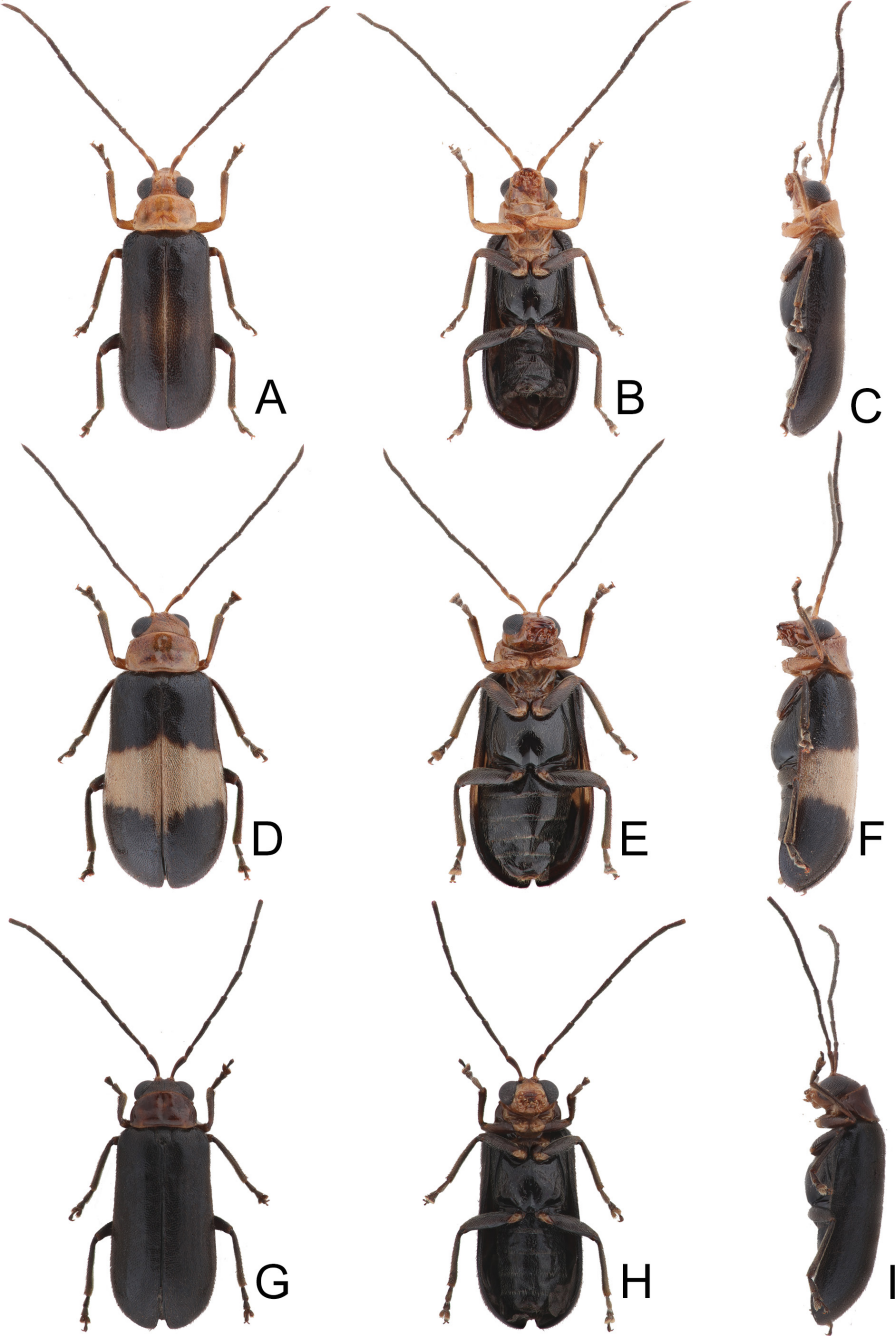
Habitus of *Anatrimonia tsoui* sp. nov. **A**. Male, dorsal view; **B**. Ditto, ventral view; **C**. Ditto, lateral view; **D**. Female, dorsal view; **E**. Ditto, ventral view; **F**. Ditto, lateral view; **G**. Male, from alpine habit (Tatachia 塔塔加), male, dorsal view; **H**. Ditto, ventral view; **I**. Ditto, lateral view.

**Figure 17.**
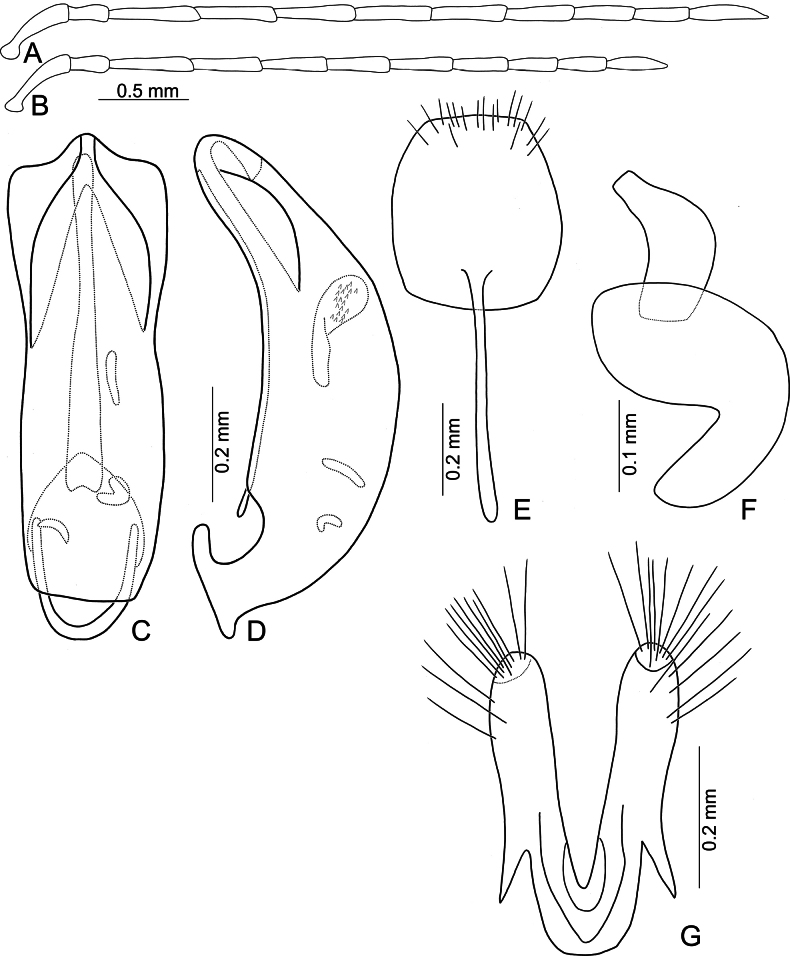
*Anatrimonia tsoui* sp. nov. **A**. Antenna, male; **B**. Antenna, female; **C**. Aedeagus, dorsal view; **D**. Aedeagus, lateral view; **E**. Abdominal ventrite VIII, female; **F**. Spermatheca; **G**. Gonocoxae.

Figs 15D, 15E, 16, 17

Trichocerophysa hainana : Kimoto 1969: 36.
Anadimonia latifascia : Kimoto 1991: 10.

#### Type specimens examined

**(*n* = 195). *Holotype*** ♂ (TARI), **Taiwan** • **Kaohsiung**: Erhchituan (二集團), 8.III.2013, leg. B.-X. Guo. ***Paratypes***. • 5♀ (TARI), same data as holotype; • 2♂ (TARI), same locality, 1.III.2009, leg. U. Ong; • **Chiayi**: 1♀ (KMNH), Alishan (阿里山), 6.VII.1965, leg. R. Ishikawa, was identified as *Trichocerophysa hainana* by Kimoto (1969); • 1♀ (TARI), same locality, 28.III.2010, leg. U. Ong; • 2♂, 2♀ (TARI), same locality, 21.VI.2014, leg. B.-X. Guo; • 3♂ (TARI), Shihcho (石卓), 20.III.2015, leg. B.-X. Guo; • 1♂, 1♀ (TARI), Tiaotiao farm (跳跳農場), 6.III.2016, leg. U. Ong; • **Hsinchu**: 1♂, 2♀ (TARI), Lupi (魯壁), 26.VII.2008, leg. M.-H. Tsou; • **Hualien**: 1♀ (TARI), Juisui trail (瑞穗林道), 5.VII.2023, leg. Y.-T. Chung; • **Kaohsiung**: 2♂ (TARI), Erhchituan (二集團), 1.IV.2015, leg. B.-X. Guo; • 1♂ (KMNH), Fengkangshan (鳳岡山), 30.IV.1986, leg. K. Baba, was identified as *Trichocerophysa hainana* and synonymized with *Anadimonia latifascia* by Kimoto (1991); • 2♀ (TARI), Tengchih (藤枝), 18.IV.2013, leg. Y.-T. Chung; • 1♂, 1♀ (TARI), Tienchih (天池), 1.IV.2015, leg. C.-F. Lee; • **Nantou**: 1♂ (TARI), Huisunlinchang (惠蓀林場), 17.X.2013, leg. F.-S. Huang; • 2♀ (TARI), same locality, 6.III.2014, leg. F.-S. Huang, 香楠 (*Machilus zuihoensis*), 噴霧 (fogging); • 3♀ (NMNS), Liyingshan (立鷹山), 28.VII.2020, leg. J.-F. Tsai; • 1♂ (KMNH), Musha (= Wushe, 霧社), 30.III.1981, leg. F. Kimura; • 1♀ (KMNH), Nanshanchi (南山溪), 26.VI.1965, leg. T. Shirôzu; • 1♀ (KMNH), same locality, 12.VII.1966, leg. H. Kamiya, both specimens collected from Nanshanchi were identified as *Trichocerophysa hainana* by Kimoto (1969); • 1♀ (KMNH), Sungkang (松崗), 4.VIII.1969, leg. T. Kobayashi; • 3♂, 1♀ (TARI), Tatachia (塔塔加), 9.VII.2014, leg. C.-L (sic!). Lee; • **Pingtung**: 1♀ (TARI), Chiamu (佳暮), 10.V.2018, leg. Y.-T. Chung; • 1♀ (TARI), Jinshuiying (浸水營), 23.VIII.2011, leg. J.-C. Chen; • 1♂, 2♀ (TARI), same locality, 12.IV.2012, leg. C.-F. Lee; • 1♂ (TARI), Machia (瑪家), 25.IX.2017, leg. Y.-T. Chung; • 1♂ (TARI), Peitawushan (北大武山), 3.VI.2014, leg. Y.-T. Chung; • 2♂, 1♀ (TARI), same but with “21.III.2015”; • 1♀ (TARI), same but with “14.VIII.2016”; • 2♀ (TARI), same but with “18.V.2025”; • 1♀ (NMNS), Shahsi Forest Road (沙溪林道), 19.IV.2009, leg. Y.-T. Chung; • 1♂, 1♀ (TARI), same locality, 20.VII.2017, leg. B.-X. Guo; • 1♀ (TARI), Shouka (壽卡), 27.II.2015; • 1♂ (TARI), T (D) ahanshan (大漢山) [= Tahanlintao (大漢林道)], 18.VII.2007, leg. C.-F. Lee; • 1♀ (TARI), same but with “20.VII.2007”; • 1♀ (TARI), same but with “26.III.2013”; • 2♂ (TARI), same but with “18.IV.2018”; • 1♂ (TARI), same locality, 4.VII.2008, leg. S.-F. Yu; • 1♂ (TARI), same locality, 18.VII.2007, leg. M.-H. Tsou; • 1♀ (TARI), same locality, 23.IX.2009, leg. J.-C. Chen; • 2♂, 1♀ (TARI), same but with “5.IV.2011”; • 1♂ (TARI), same but with “10.IV.2023”; • 1♂, 2♀ (TARI), same locality, 14.VIII.2011, leg. Y.-T. Wang; • 1♂, 2♀ (TARI), same locality, 25.III.2013, leg. B.-X. Guo; • 1♀ (TARI), same but with “30.VII.2013”; • 1♀ (TARI), same but with “9.VIII.2013”; • 1♂, same locality, 30.VII.2012, leg. Y.-T. Chung; • 1♀ (TARI), same but with “3.IX.2012”; • 2♀ (TARI), same but with “3.IV.2013”; • 1♀ (TARI), same but with “16.IV.2013”; • 1♀ (TARI), same but with “24.IV.2013”; • 1♀ (TARI), same but with “21.VII.2013”; • 3♂, 6♀ (TARI), same but with “30.VII.2013”; • 1♀ (TARI), same but with “2.IX.2013”; • 1♂ (TARI), same but with “6.IV.2014”; • 1♀ (TARI), same but with “26.VII.2014”; • 1♀ (TARI), same but with “28.III.2015”; • 3♂, 2♀ (TARI), same but with “2.IV.2015”; • 1♂ (TARI), same but with “8.IV.2016”; • 1♀ (TARI), same but with “10.V.2016”; • 1♂ (TARI), same but with “17.VI.2016”; • 3♂, 3♀ (TARI), same but with “28.VII.2016”; • 1♂, 2♀ (TARI), same but with “6.VIII.2016”; • 1♂, 2♀ (TARI), same but with “22.VIII.2016”; • 4♂ (TARI), same but with “4.IV.2017”; • 7♂, 2♀ (TARI), same but with “10.IV.2017”; • 4♀ (TARI), same but with “17.IV.2017”; • 2♀ (TARI), same but with “22.IV.2017”; • 4♂, 1♀ (TARI), same but with “9.IV.2018”; • 1♂, 2♀ (TARI), same but with “15.IV.2018”; • 2♀ (TARI), same but with “23.IV.2018”; • 2♀ (TARI), same but with “23.IX.2019”; • 1♀ (TARI), same but with “20.VII.2020”; • 1♀ (TARI), same but with “23.VII.2020”; • 1♀ (TARI), same but with “5.VIII.2020”; • 2♀ (TARI), same but with “12.IV.2021”; • 1♀ (TARI), same but with “7.VIII.2022”; • 1♀ (TARI), same but with “25.IV.2023”; • 1♀ (TARI), same locality, 6.IV.2013, leg. W.-C. Liao; • 1♂ (TARI), same locality, 19.VII.2013, leg. M.-H. Tsou; • 1♀ (TARI), same locality, 15.III.-3.IV.2020, Malaise trap, leg. Y.-C. Chiu; • 1♀ (TARI), same but with “2.V.-6.VI.2020”; • 1♀ (TARI), same but with “6.VI.-25.VI.2020”; • 1♀ (TARI), Peitawushan (北大武山), 18.VI.2012, leg. J.-C. Chen; • 1♂, 1♀ (TARI), same but with “14.VI.2022”; • 2♂ (TARI), same but with “23.III.2024”; • 3♂ (TARI), same but with “13.III.2025”; • 2♂ (TARI), same but with “20.III.2025”; • 1♂ (TARI), same locality, 11.VIII.2013, leg. Y.-T. Chung; • 1♂ (TARI), same but with “15.VIII.2013”; • 1♀ (TARI), same but with “9.V.2024”; • 1♂ (TARI), same but with “23.VII.2025”; • 1♀ (TARI), Wutai (霧台), 12.V.2009, leg. U. Ong; • 1♂ (TARI), same locality, 8.V.2025, leg. Y.-T. Chung; • **Tainan**: 1♀ (TARI), Meiling (梅嶺), 11.V.2014, leg. B.-X. Guo; • 3♂, 2♀ (TARI), same but with “28.II.2016”; • **Taipei**: 3♂ (TARI), Fengkueitsui (風櫃嘴), 26.VIII.2007, leg. M.-H. Tsou; • 2♀ (TARI), Tatunshan (大屯山), 23.IV.2016, leg. Y.-F. Hsu; • **Taitung**: 1♀ (TARI), Hsiangyang (向陽), 23.VI.2010, leg. M.-H. Tsou; • 2♂, 2♀ (TARI), same locality, 18.VII.2014, leg. W.-C. Huang; • 1♂, 1♀ (TARI), Liyuan (栗園), 19.VI.2013, leg. C.-F. Lee; • 2♀ (TARI), same but with “24.VII.2013”; • 1♂ (TARI), Tulanshan (都蘭山), 4.VI.2010, leg. J.-C. Chen.

#### Diagnosis.

**Figure 18.**
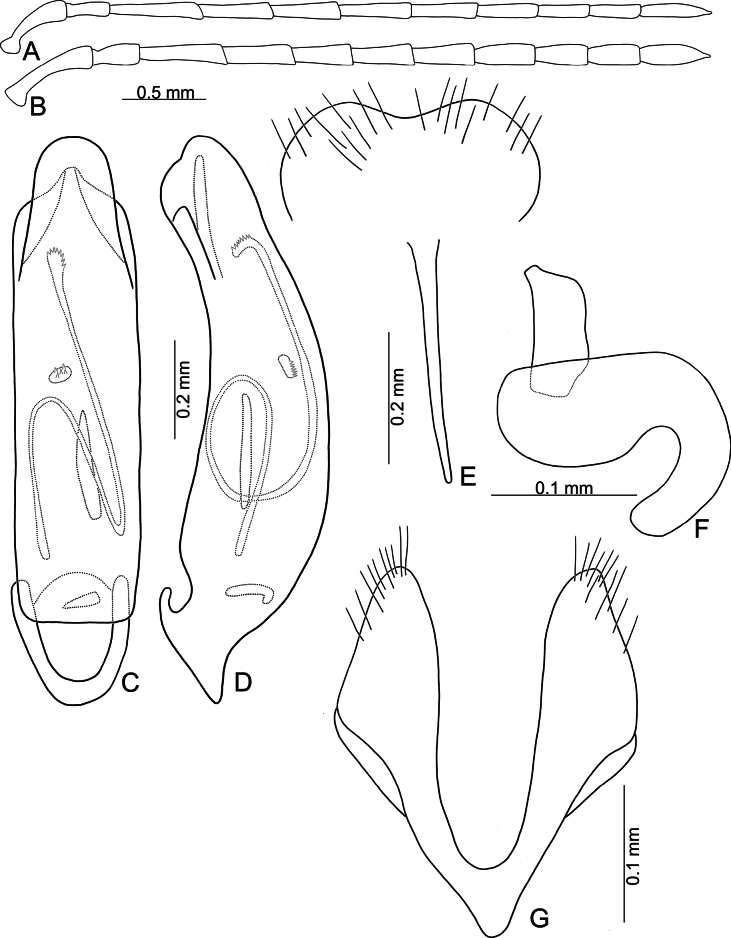
*Anatrimonia wangi* sp. nov. **A**. Antenna, male; **B**. Antenna, female; **C**. Aedeagus, dorsal view; **D**. Aedeagus, lateral view; **E**. Abdominal ventrite VIII, female; **F**. Spermatheca; **G**. Gonocoxae.

Adults of *Anatrimonia tsoui* sp. nov. are similar to those of *A. albofasciata* (Jacoby, 1892), *A. cheni* sp. nov., *A. hainana* (Gressitt & Kimoto, 1963), and *A. wangi* sp. nov. in possessing yellowish heads and prothoraxes, black elytra, and middle and hind legs. However, the median transverse white bands on the elytra are present only in females of *A. tsoui* sp. nov. (Fig. 16A–F) [median transverse white bands on elytra present in both sexes of other species]. In addition, the aedeagus of *A. tsoui* sp. nov. is diagnostic: dorsal ridges not projecting from apex of aedeagus (Fig. 17C, D) [dorsal ridges projecting from apex of aedeagus in *A. wangi* sp. nov. (Fig. 18C, D)], base of dorsal ridges narrower than aedeagus (Fig. 17C) [base of dorsal ridges wider than aedeagus in *A. albofasciata* (Fig. 5C) and *A. hainana* (Fig. 9C)], dorsal ridges moderately and medially rounded (Fig. 17C) [dorsal ridges recurved near base in *A. cheni* sp. nov. (Fig. 7C)], endophallic sac composed of three small sclerites (Fig. 17C, D) [one flagellum-like endophallic sclerites, and two or three small sclerites in *A. cheni* sp. nov. (Fig. 7C, D) and *A. wangi* sp. nov. (Fig. 18C, D)].

#### Description.

Length 4.1–6.8 mm, width 1.7–2.7 mm. General color black (Fig. 16A–F); head and prothorax pale yellow, antenna blackish-brown, but antennomeres I and II, and base of antennomere III paler; middle and hind legs black, but trochanters yellowish-brown, front legs pale yellow, but apical 2/3 of tibia, and tarsi dark brown; each elytron with one transverse white band at middle in most females, but reduced in all males and some females. Antennae (Fig. 17A) filiform in males, ratio of lengths of antennomeres I–XI 1.0 : 0.5 : 1.1 : 1.0 : 1.0 : 1.0 : 1.0 : 0.9 : 0.8 : 0.7 : 1.0; ratio of length to width of antennomeres I–XI 4.1 : 2.5 : 5.8 : 5.2 : 5.2 : 5.0 : 4.9 : 4.4 : 4.2 : 4.1 : 5.6; shorter in females, ratio of lengths of antennomeres I–XI (Fig. 18B) 1.0 : 0.5 : 1.0 : 0.9 : 0.8 : 0.8 : 0.8 : 0.7 : 0.6 : 0.6 : 0.8; ratio of length to width of antennomeres I–XI 4.4 : 2.5 : 5.7 : 4.8 : 4.6 : 4.4 : 4.4 : 3.9 : 3.4 : 3.4 : 4.5. Pronotum 1.70–1.77 × wider than long; disc with sparse, coarse and fine punctures at sides; lateral margins slightly rounded and basally narrowed, basal margin and apical margin straight; disc with a broad transverse depression at each side, median longitudinal groove obvious. Elytra 1.78–1.93 × longer than wide; with extremely dense setae, parallel sided, apex convergent. Aedeagus (Fig. 18C, D) broad, ~ 3.3 × longer than wide; lateral margins narrowed at apical 2/5, apical margin truncate, slightly convex at middle; apico-lateral margins recurved; dorsal disc with reverse V-shaped ridge from near apex to apical 2/5; strongly curved in lateral view; endophallic sac with three small sclerites, anterior sclerites laterally flattened and with dense, tiny teeth. Gonocoxae (Fig. 18G) cylindrical, apex widely rounded; each gonocoxa with 10–13 long setae at apex, both gonocoxae basally connected with each other. Ventrite VIII (Fig. 18E) weakly sclerotized, apical margin truncate; disc with sparse, short setae near apical margin; spiculum slender and long. Spermathecal receptaculum (Fig. 18F) moderately swollen; pump wide and curved; sclerotized spermathecal duct wide but short.

#### Variation.

Specimens collected from high altitudes (> 2500 m) have blackened bodies.

#### Food plants.

Lauraceae: *Litsea acuminata* (Blume) Kurata, *L. akoensis* Hayata, *L. elongata* var. *mushaensis* (Hayata) J.C. Liao (Fig. 15E), *Machilus philippinensis* Merr., *M. thunbergii* Siebold & Zucc. (Fig. 15D), *M. zuihoensis* Hayata.

#### Etymology.

This new species is named for Mei-Hua Tsou (曹美華), the first member of TCRT to collect specimens of this new species.

#### Distribution.

This species is widespread in Taiwan and is the most common species in the genus.

### Anatrimonia wangi sp. nov.

Taxon classificationAnimaliaColeopteraChrysomelidae

C534C87F-BDC0-520A-A03B-2C74CA98745F

https://zoobank.org/DB220CD4-9E92-46D0-B335-7EEF1C34DC9D

Fig. 18

#### Type specimens examined

**(*n* = 2). *Holotype*** ♂ (TARI), **China** • **Hainan**: Mingfengku (鳴鳳谷), 13.XI.2018, leg. Y.-T. Wang. ***Paratype*. China** • Hainan: 1♀ (TARI), Jianfengling (尖峰嶺), 10.V.2015, leg. Y.-T. Wang.

#### Diagnosis.

Adults of *Anatrimonia wangi* sp. nov. are similar to those of *A. albofasciata* (Jacoby), *A. cheni* sp. nov., and *A. hainana* (Gressitt & Kimoto) in possessing yellowish heads and prothoraxes, and black elytra with median transverse white bands in both sexes. However, adults of *A. wangi* sp. nov. and *A. hainana* (Fig. 4D–F) have black antenna [yellow antenna in *A. albofasciata* (Fig. 4A–C) and *A. cheni* sp. nov. (Fig. 6A–C)]. Adults of *A. wangi* sp. nov. have yellow scutellum and abdomen (black scutellum and abdomen in *A. hainana*)], aedeagus with dorsal ridge projecting anteriorly from apex of aedeagus (Fig. 18C, D) [aedeagus with dorsal ridge, but not projecting anteriorly from apex of aedeagus in *A. hainana* (Fig. 9C, D)], endophallic sac composed of one extremely slender, flagellum like sclerite and three small ones (Fig. 18C, D) [three small endophallic sclerites in *A. hainana* (Fig. 9C, D)].

#### Description.

Length 4.5–5.7 mm, width 1.9–2.1 mm. General color black; head, prothorax, scutellum, and abdomen pale yellow, antenna blackish-brown except for three basal antennomeres; middle and hind legs black but trochanters yellowish-brown, front legs pale yellow, but apical 2/3 of tibia, and tarsi dark brown; each elytron with one transverse white band at middle. Antennae (Fig. 18A) filiform in males, ratio of lengths of antennomeres I–XI 1.0 : 0.6 : 1.2 : 1.0 : 0.9 : 0.9 : 0.9 : 0.8 : 0.7 : 0.7 : 0.9; ratio of length to width of antennomere I–XI 3.8 : 3.2 : 5.1 : 4.6 : 4.0 : 4.4 : 4.1 : 4.2 : 3.9 : 3.9 : 5.3; wider in females, ratio of lengths of antennomeres I–XI (Fig. 18B) 1.0 : 0.5 : 1.2 : 1.0 : 0.9 : 0.9 : 0.8 : 0.8 : 0.7 : 0.7 : 0.9; ratio of length to width of antennomeres I–XI 4.2 : 2.8 : 5.4 : 4.7 : 4.4 : 4.8 : 4.2 : 4.1 : 3.7 : 3.6 : 5.0. Pronotum 1.53–1.57 × wider than long; disc with sparse, fine punctures at sides; lateral margins slightly rounded and basally narrowed, basal margin straight, apical margin slightly and medially convex; disc with a broad transverse depression at each side, median longitudinal groove obvious. Elytra 1.80–1.91 × longer than wide; with extremely dense setae, parallel sided, apex convergent. Aedeagus (Fig. 18C, D) slender, ~ 4.6 × longer than wide; parallel-sided; apical margin convex at middle; apico-lateral margins recurved; dorsal disc with square sclerite projecting from apex; endophallic sac with four sclerites, one sclerite flagellum-like, apex with teeth, a second smaller, flattened with several teeth, a third transverse, and a fourth slender. Gonocoxae (Fig. 18G) cylindrical but basally wider, apex widely rounded; each gonocoxa with 10–11 short setae at apex, both gonocoxae basally connected with one slender sclerite, base triangular. Ventrite VIII (Fig. 18E) well sclerotized, apical margin moderately concave at middle; disc with sparse, short setae near apical and apico-lateral margin; spiculum slender and long. Spermathecal receptaculum (Fig. 18F) moderately swollen; pump wide and curved; sclerotized spermathecal duct wide but short.

#### Food plants.

Unknown.

#### Etymology.

The new species is named after Mr. Yu-Tung Wang (王宇堂) who collected specimens of this new species.

#### Distribution.

China (Hainan).

### Anatrimonia yuae sp. nov.

Taxon classificationAnimaliaColeopteraChrysomelidae

CB2DB84B-13EE-58D6-87B4-E2B759EF43AB

https://zoobank.org/89970CB1-2742-48B6-9BD2-149F7E127779

Figs 12D–F, 15F, 19

#### Type specimens examined

**(*n* = 5). *Holotype*** ♂ (TARI), **Taiwan** • **Nantou**: Tatachia (塔塔加), 21.VII.2009, leg. S.-F. Yu. ***Paratypes***. • 1♂, 3♀ (TARI), same data as holotype.

#### Diagnosis.

**Figure 19.**
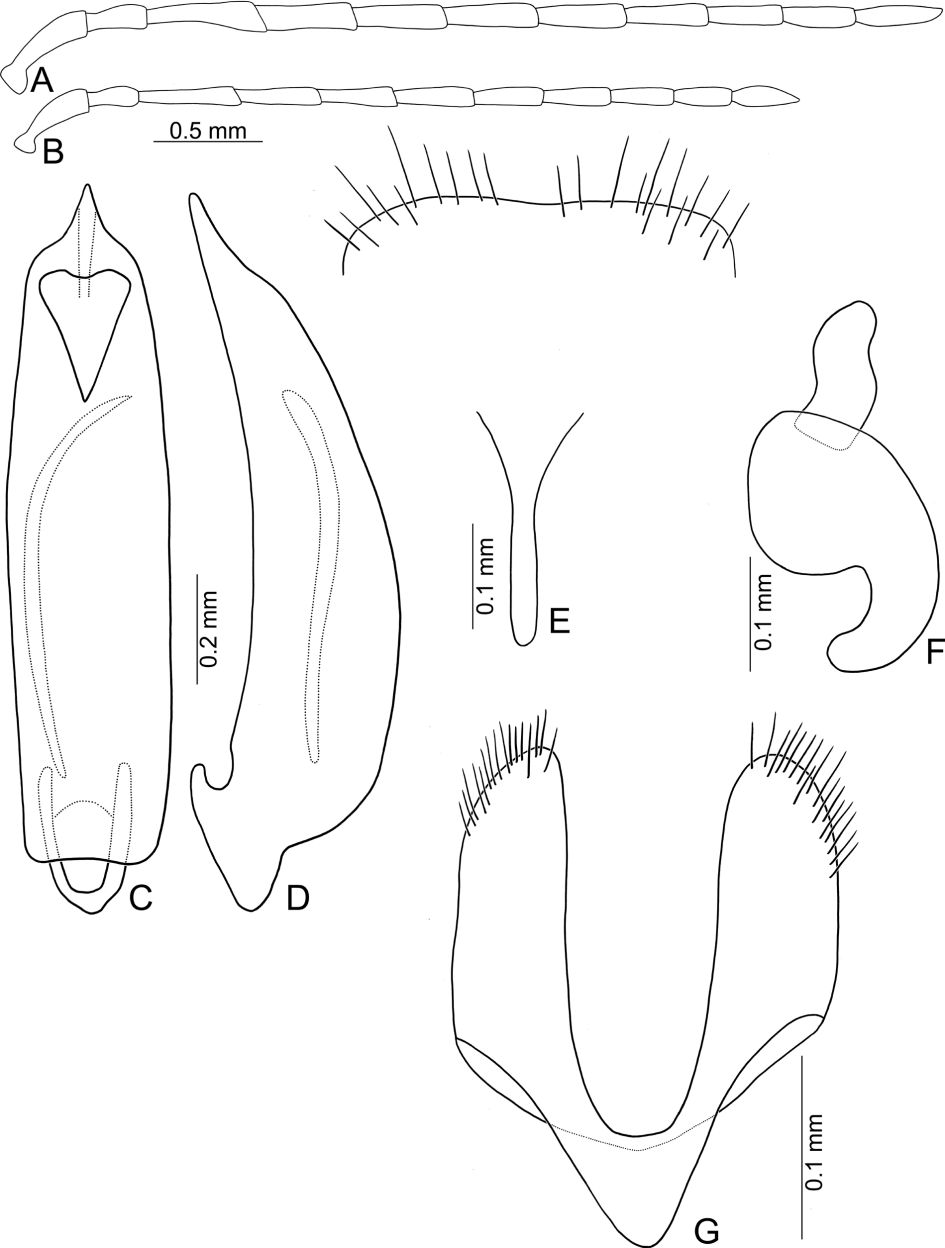
*Anatrimonia yuae* sp. nov. **A**. Antenna, male; **B**. Antenna, female; **C**. Aedeagus, dorsal view; **D**. Aedeagus, lateral view; **E**. Abdominal ventrite VIII, female; **F**. Spermatheca; **G**. Gonocoxae.

**Figure 20.**
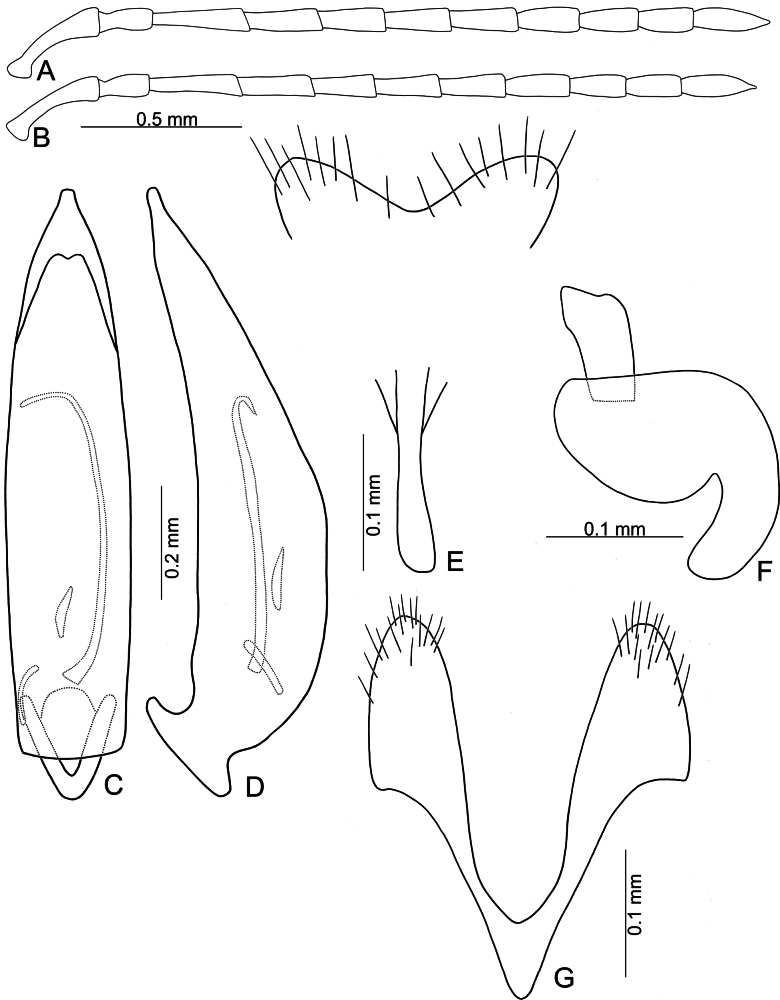
*Anatrimonia yunnanensis* sp. nov. **A**. Antenna, male; **B**. Antenna, female; **C**. Aedeagus, dorsal view; **D**. Aedeagus, lateral view; **E**. Abdominal ventrite VIII, female; **F**. Spermatheca; **G**. Gonocoxae.

Adults of *Anatrimonia yuae* sp. nov., *A. jungchani* sp. nov., and *A. yunnanensis* sp. nov. are characterized by their yellow elytra (Fig. 12). Adults of *A. yuae* sp. nov. are similar to those of *A. jungchani* sp. nov. with large body sizes (> 4.3 mm) and yellow metathoracic ventrites (Fig. 12A–F) in contrast to the small body sizes (< 4.3 mm) and black metathoracic ventrites in *A. yunnanensis* sp. nov. (Fig. 12G–I). The aedeagi of these species are diagnostic: strongly and apically narrowed but with acute apex in *A. yuae* sp. nov. (Fig. 19C, D), gradually and apically narrowed but narrowly rounded apex in *A. jungchani* sp. nov. (Fig. 13C, D), and strongly and apically narrowed but with truncate apex in *A. yunnanensis* sp. nov. (Fig. 20C, D); endophallic sac composed of only one slender endophallic sclerite in *A. yuae* sp. nov. (Fig. 19C, D), one slender endophallic sclerites and two small sclerites in *A. jungchani* sp. nov. (Fig. 13C, D) and *A. yunnanensis* sp. nov. (Fig. 20C, D).

#### Description.

Length 4.7–4.8 mm, width 1.8–2.1 mm. General color pale yellow (Fig. 12D–F); metasternum and abdomen dark brown. Antennae (Fig. 19A) filiform in males, ratio of lengths of antennomeres I–XI 1.0 : 0.6 : 1.1 : 0.9 : 0.9 : 0.9 : 0.8 : 0.8 : 0.7 : 0.7 : 0.9; ratio of length to width of antennomeres I–XI 3.8 : 2.4 : 3.8 : 3.6 : 3.8 : 4.1 : 3.8 : 3.8 : 3.6 : 3.5 : 4.6; smaller in females, ratio of lengths of antennomeres I–XI (Fig. 19B) 1.0 : 0.6 : 1.1 : 1.0 : 0.9 : 0.9 : 0.8 : 0.8 : 0.7 : 0.7 : 0.8; ratio of length to width of antennomeres I–XI 3.5 : 2.7 : 5.0 : 4.3 : 3.9 : 3.9 : 3.8 : 3.6 : 3.4 : 3.4 : 3.4. Pronotum 1.53–1.63 × wider than long; disc with sparse, fine punctures at sides; lateral margins slightly rounded and basally narrowed, some × straight, apical and basal margins straight; disc with a broad transverse depression at each side, median longitudinal groove obvious. Elytra 1.78–2.02 × longer than wide; with extremely dense setae, parallel-sided, apex convergent. Aedeagus (Fig. 19C, D) narrow, ~ 4.5 × longer than wide; parallel-sided, apex lanceolate, strongly subapically narrowed; dorsal disc without ridges apically; slightly curved in lateral view; endophallic sac with one slender, longitudinal sclerite. Gonocoxae (Fig. 19G) cylindrical, apex widely rounded; each gonocoxa with 16 or 17 short setae at apex, both gonocoxae basally connected with narrow sclerite, connection triangular. Ventrite VIII (Fig. 19E) well sclerotized, apical margin subtruncate, slightly depressed at middle; disc with sparse, long setae near apical margin; spiculum slender and short. Spermathecal receptaculum (Fig. 19F) moderately swollen; pump wide and curved; sclerotized spermathecal duct wide but short.

#### Food plants.

Lauraceae: *Litsea hypophaea* Hayata (Fig. 15F).

#### Etymology.

This new species is named for Su-Fang Yu (余素芳), the first member of TCRT to collect specimens of this new species.

#### Distribution.

The species is only known from the type locality.

### Anatrimonia yunnanensis sp. nov.

Taxon classificationAnimaliaColeopteraChrysomelidae

80D826E0-201D-57FC-BD51-7B6DD6E4CFBB

https://zoobank.org/DA5B9D88-3288-4A15-B708-6308B44725DA

Figs 12G–I, 20

#### Type specimens examined

**(*n* = 27). *Holotype*** ♂ (TARI), **China** • **Yunnan**: Wuzhidong (五之洞), 12.IX.2015, leg. Y.-T. Wang. ***Paratypes***. • 6♂, 8♀ (TARI), same data as holotype; 3♂, 5♀, same but with “14.IX.2015; **China** • Yunnan: 1♂, 2♀ (TARI), Bangda (邦達村), 8.X.2015, leg. Y.-T. Wang; 1♀ (TARI), Banggunjianshan (幫棍尖山), 20.V.2016, leg. Y.-T. Wang; **Thailand** • Mae Hong Son: 1♀ (JBCB), SE of Soppong, 1500 m, 19°27'N, 98°20'E, 23–27.V.1999, leg. M. Říha.

#### Diagnosis.

Adults of *Anatrimonia yunnanensis* sp. nov., *A. jungchani* sp. nov., and *A. yuae* sp. nov. are characterized by their yellow elytra (Fig. 12). Adults of *A. yunnanensis* sp. nov. differ from those of *A. yuae* sp. nov., and *A. jungchani* sp. nov. in having smaller body sizes (< 4.3 mm) and black metathoracic ventrites (Fig. 12G–I) in contrast to larger body sizes (> 4.3 mm) and yellow metathoracic ventrites of *A. yuae* sp. nov. and A. *jungchani* sp. nov. (Fig. 12A–F). The aedeagi of these species are diagnostic: strongly and apically narrowed but with truncate apex in *A. yunnanensis* sp. nov. (Fig. 20C, D), gradually and apically narrowed but narrowly rounded apex in *A. jungchani* sp. nov. (Fig. 13C, D), and strongly and apically narrowed but with acute apex in *A. yuae* sp. nov. (Fig. 19C, D); endophallic sac composed of one slender endophallic sclerites and two small sclerites in *A. yunnanensis* sp. nov. (Fig. 20C, D) and *A. jungchani* sp. nov. (Fig. 13C, D), only one slender endophallic sclerite in *A. yuae* sp. nov. (Fig. 19C, D).

#### Description.

Length 3.5–4.2 mm, width 1.6–1.9 mm. General color pale yellow (Fig. 12G–I); antennae blackish-brown except for three basal antennomeres; legs yellowish-brown, outer margin of tibia darker; metathoracic ventrites black. Antennae (Fig. 20A) filiform in males, ratio of lengths of antennomeres I–XI 1.0 : 0.5 : 0.9 : 0.7 : 0.6 : 0.6 : 0.7 : 0.6 : 0.6 : 0.5 : 0.8; ratio of length to width of antennomeres I–XI 3.5 : 2.5 : 4.0 : 3.2 : 2.8 : 2.8 : 3.0 : 2.7 : 2.5 : 2.4 : 3.5; similar in females, ratio of lengths of antennomeres I–XI (Fig. 20B) 1.0 : 0.5 : 0.9 : 0.7 : 0.6 : 0.6 : 0.6 : 0.6 : 0.5 : 0.5 : 0.7; ratio of length to width of antennomeres I–XI 4.1 : 2.2 : 3.9 : 3.3 : 2.8 : 2.8 : 2.9 : 2.9 : 2.5 : 2.5 : 3.4. Pronotum 1.73–1.79 × wider than long; disc with sparse, coarse and fine punctures at sides; lateral margins slightly rounded and basally narrowed, basal margin and apical margin straight; disc with a broad transverse depression at each side, median longitudinal groove obvious. Elytra 1.52–1.68 × longer than wide; with extremely dense setae, parallel sided, apex convergent. Aedeagus (Fig. 20C, D) slender, ~ 4.7 × longer than wide; lateral margins parallel from base to middle, apically narrowed from middle, apex tubular, apical margin narrowly rounded; slightly and curved in lateral view; endophallic sac with one flagellum-like sclerite, apically abbreviated, basal margin truncate, with two additional slender sclerites near base of flagellum-like sclerite. Gonocoxae (Fig. 20G) short and broad, apically narrowed, apex widely rounded; each gonocoxa with 14 or 15 short setae at apex, both gonocoxae basally conjunct with one extremely slender and longitudinal sclerite, base triangular. Ventrite VIII (Fig. 20E) well sclerotized, median depression at middle of apical margin; disc with sparse, short setae near apical margin; spiculum slender but short. Spermathecal receptaculum (Fig. 20F) moderately swollen; pump wide and curved; sclerotized spermathecal duct wide but short.

#### Food plants.

Unknown.

#### Etymology.

This new species is named for the type locality.

#### Distribution.

China (Yunnan).

##### Key to species of *Anatrimonia*

**Table:** 

1	Elytra entirely yellow (Fig. 12)	**2**
–	black, some × with median transverse white band on each elytron (Figs 6, 10, 16)	**4**
2	body size (> 4.3 mm); metathoracic ventrite yellow (Fig. 12B, E)	**3**
–	Small body size (< 4.3 mm); metathoracic ventrite black (Fig. 2H)	***A. yunnanensis* sp. nov**.
3	gradually and apically narrowed; endophallic sac with 1 flagellum-like sclerite and 2 small sclerites (Fig. 13C, D)	***A. jungchani* sp. nov**.
–	strongly and apically narrowed; endophallic sac with 1 flagellum-like sclerite (Fig. 19C, D)	***A. yuae* sp. nov**.
4	and prothorax red (Fig. 10)	**5**
–	and prothorax yellow (Figs 6, 16)	**6**
5	elytra without median transverse white bands (Fig. 12G–I); apex of dorsal ridges widely rounded and not projected from aedeagus; endophallic sac with 3 small sclerites (Fig. 14C, D)	***A. meihuai* sp. nov**.
–	elytra with more or less transverse white bands (Fig. 12A–F); apex of dorsal ridges narrowly rounded and projected from aedeagus; endophallic sac with 1 flagellum-like sclerite, 1 slender sclerite, and 1 small sclerite (Fig. 11C, D)	***A. huangi* sp. nov**.
6	legs yellow (Fig. 6D–F); aedeagus without apico-lateral expansion and dorsal ridges (Fig. 8C, D)	***A. cheni* sp. nov**.
–	and hind legs black (Fig. 6A–C); aedeagus with apico-lateral expansion and dorsal ridges (Figs 5C, 5D; 7C, D; 9C, D; 17C. D; 18C, D)	**7**
7	transverse white bands on elytra absent in males but present only in most females, distributed in Taiwan (Fig. 16A–F)	***A. tsoui* sp. nov**.
–	transverse white bands on elytra present in both sexes, distributed in China, Southeast Asia	**8**
8	black, distributed in Hainan (China) (Fig. 4D–F)	**9**
–	yellow, distributed in other parts of China and Southeast Asia	**10**
9	and abdomen black; dorsal ridges not projected from aedeagus, endophallic sac with 3 small sclerites (Fig. 9C, D)	***A. hainana* (Gressitt & Kimoto)**
–	and abdomen yellow; dorsal ridges projected from aedeagus; endophallic sac with 1 flagellum-like sclerite and 3 small sclerites (Fig. 18C, D)	***A. wangi* sp. nov**.
10	IV–X without longitudinal ridges in males; base of dorsal ridges wider than aedeagus; endophallic sac with three small sclerites (Fig. 5C, D)	***A. albofasciata* (Jacoby)**
–	IV–X with longitudinal ridges in males; base of dorsal ridges narrower than aedeagus; endophallic sac with 1 flagellum-like sclerite and 2 small sclerites (Fig. 7C, D)	***A. cheni* sp. nov**.

## Discussion

The food plant of *Anadimonia potanini* is unknown. Adults of *Anatrimonia* gen. nov. are not monophagous but oligophagous for Lauraceae. They can be collected by sweeping leaves of Lauraceae with extendable insect nets. Although some specimens in the historical collection at the Taiwan Agricultural Research Institute were collected below 2 m height by sweeping, no adults of *Anatrimonia* gen. nov. were found during recent collecting at this heigh. Specimens were collected between 3–6 m by members of TCRT. For some species such as *A. tsoui* sp. nov., they were easily collected this way, although few adults were collected during each collecting trip. Adults appeared during March to October each year based on these colleting records. Fogging programs may be the best way to collect adults of this genus. For example, fogging programs were conducted nine × on *Machilus zuihoensis* on 26 August 2013 and 10 July 2014. Adults of *A. meihuai* sp. nov. and *A. tsoui* sp. nov. were collected on 17 October 2013, 6 March 2014, 3 April 2014, and 12 June 2014. Three of five events produced no adults of *Anatrimonia* gen. nov. during winter (4 December 2013, 19 December 2013, and 16 January 2014). Fogging was conducted four × on *Neolitsea variabillima* (Hayata) during 2013–2014. Adults of *A. huangi* sp. nov. were collected on 16 September 2013 and 3 July 2014. No adults were collected on 9 January 2014 and 27 March 2014 during winter inactivity.

The results indicate that six species of *Anatrimonia* gen. nov. occur in Taiwan and five additional species occur in China and Southeast Asia, where species diversity is likely underestimated. Although adults can be collected by sweeping with extendable insect nets, this is not an efficient method since few adults of most species were collected. Fogging programs are recommended as a method to collect adults to better understand diversity of *Anatrimonia* gen. nov. in China and Southeast Asia.

## Supplementary Material

XML Treatment for Anadimonia

XML Treatment for Anadimonia potanini

XML Treatment for Anatrimonia

XML Treatment for Anatrimonia albofasciata

XML Treatment for Anatrimonia cheni

XML Treatment for Anatrimonia chungi

XML Treatment for Anatrimonia hainana

XML Treatment for Anatrimonia huangi

XML Treatment for Anatrimonia jungchani

XML Treatment for Anatrimonia meihuai

XML Treatment for Anatrimonia tsoui

XML Treatment for Anatrimonia wangi

XML Treatment for Anatrimonia yuae

XML Treatment for Anatrimonia yunnanensis

## Additional information

### Use of AI

No use of AI was reported.

### Author ORCIDs

Chi-Feng Lee https://orcid.org/0000-0003-1996-0557

Jan Bezděk https://orcid.org/0000-0003-4358-7211

### Data availability

All of the data that support the findings of this study are available in the main text.
